# Silencing susceptibility genes in potato hinders primary infection with *Phytophthora infestans* at different stages

**DOI:** 10.1093/hr/uhab058

**Published:** 2022-01-19

**Authors:** Kaile Sun, Danny Schipper, Evert Jacobsen, Richard G F Visser, Francine Govers, Klaas Bouwmeester, Yuling Bai

**Affiliations:** 1 College of Horticulture, Henan Agricultural University, Nongye Road 63, 450002 Zhengzhou, Henan, China; 2 Plant Breeding, Wageningen University & Research, Droevendaalsesteeg 1, 6708 PB Wageningen, The Netherlands; 3 Laboratory of Phytopathology, Wageningen University & Research, Droevendaalsesteeg 1, 6708 PB, Wageningen, The Netherlands; 4 Biosystematics Group, Wageningen University & Research, Droevendaalsesteeg 1, 6708 PB Wageningen, The Netherlands

## Abstract

Most potato cultivars are susceptible to late blight disease caused by the oomycete pathogen *Phytophthora infestans*. Here we report that the genetic loss of host susceptibility is a new source of resistance to prevent or diminish pathogen infection. Previously, we showed that RNAi-mediated silencing of the potato susceptibility (*S*) genes *StDND1*, *StDMR1*, and *StDMR6* leads to increased late blight resistance. The mechanisms underlying this *S*-gene-mediated resistance have thus far not been identified. In this study, we examined the infection process of *P. infestans* in *StDND1*-, *StDMR1*-, and *StDMR6-*silenced potato lines. Microscopic analysis showed that penetration of *P. infestans* spores was hampered in *StDND1*-silenced plants. In *StDMR1-* and *StDMR6-*silenced plants, *P. infestans* infection was arrested at a primary infection stage by enhanced cell death responses*.* Histochemical staining revealed that *StDMR1-* and *StDMR6-*silenced plants display elevated ROS levels in cells at the infection sites. Resistance in *StDND1*-silenced plants, however, seems not to rely on a cell death response as ROS accumulation was found to be absent at most inoculated sites. Quantitative analysis of marker gene expression suggests that the increased resistance observed in *StDND1*- and *StDMR6*-silenced plants relies on an early onset of salicylic acid- and ethylene-mediated signaling pathways. Resistance mediated by silencing *StDMR1* was found to be correlated with the early induction of salicylic acid-mediated signaling. These data provide evidence that different defense mechanisms are involved in late blight resistance mediated by functional impairment of different potato *S*-genes.

## Introduction

Late blight, caused by the oomycete *Phytophthora infestans*, is one of the most devastating diseases of potato worldwide. The pathogen produces sporangia, asexual spores that spread by wind and rain. Infection starts when a sporangium that has landed on a leaf germinates directly, or develops into a zoosporangium releasing zoospores that encyst and germinate. Thereafter, the emerging germ tube develops into an appressorium that penetrates the leaf cuticle and epidermis. Infection expands throughout the leaf tissue via hyphal growth, resulting in water-soaked lesions that turn black. After a few days, the infected leaf tissue becomes necrotic and starts to sporulate. Depending on environmental conditions, an unprotected potato field can be devastated within 10 days [1].

A strategy to control late blight is to grow resistant potato cultivars obtained by introgressing disease resistance (*R*) genes that are effective against diverse *P. infestans* isolates [1]. More than 35 potato *R* genes conferring late blight resistance have been identified to date, most of which encode nucleotide-binding domain, leucine-rich repeat-containing (NLR) proteins [2–6]. These intracellular proteins mount successive defense responses when they recognize corresponding avirulence (AVR) proteins secreted by the pathogen. These AVR proteins, known as RXLR effectors, display high evolutionary rates, and as a result *P. infestans* can rapidly escape NLR-mediated resistance, thereby limiting the durability of genetic resistance in cultivars possessing a single *R* gene [7, 8]. Stacking of *R* genes is a potential strategy to achieve broad-spectrum and durable late blight resistance [9], as shown in cultivar ‘Sarpo Mira’, which has at least five introgressed *R* genes (i.e. *R3a*, *R3b*, *R4*, *Rpi-Smira1*, and *Rpi-Smira2*) from different genetic sources [10]. There is still a risk that such *R*-gene-rich potato varieties are overcome by the pathogen if no regional resistance gene strategy is developed [10]. Next to *R*-gene-mediated resistance, infection by *P. infestans* can be halted by enhancing the overall host defense status, for example through transcriptional activation of defense-related genes, accumulation of defense metabolites and cell wall reinforcement [11–14].

Another approach to gaining disease resistance is impairment of plant susceptibility genes (*S* genes), a concept that has been exploited in the last decade to develop novel breeding strategies to control diverse crop diseases [15–17]. More than 150 *S* genes have been described in *Arabidopsis*, and there is increasing evidence that *S*-gene orthologs are present in diverse crop species [18–20]. In potato, for example, reduced expression via RNAi-mediated silencing of the *S* gene *SYNTAXIN-RELATED1* (*StSYR1*), an ortholog of *AtPEN1* in *Arabidopsis*, decreased susceptibility to *P. infestans* [21].

In our previous studies, we selected 11 *Arabidopsis thalianaS* genes and silenced their orthologs in the late blight-susceptible potato cultivar (cv.) ‘Désirée’. RNAi-mediated silencing of six of these genes, which included *StDND1*, *StDMR1*, and *StDMR6*, resulted in increased resistance to *P. infestans* [15, 18]. This showed that loss of function of a putative *S* gene can be exploited to hamper infection and leaf colonization by *P. infestans*, and can generate late blight resistance in potato.

*StDND1*, *StDMR1*, and *StDMR6* are the orthologs of *Arabidopsis DND1*, *DMR1*, and *DMR6*, respectively. *Arabidopsis DND1* (*Defense, No Death 1*) encodes a cyclic nucleotide-gated cation channel (CNGC) and the *dnd1* mutant showed resistance to the bacterium *Pseudomonas syringae* pv. *glycinea* and the oomycete *Hyaloperonospora arabidopsidis*, as well as the fungal pathogens *Botrytis cinerea* and *Alternaria brassicicola* [22–27]. As indicated by the gene name “*Defense, No Death*”, resistance to *P. syringae* observed in the *dnd1* mutant was not associated with a hypersensitive response [28], but did show a constitutively elevated expression level of the pathogenesis-related gene *PR1* [23, 28]. Our previous results showed that silencing of *DND1* orthologs in tomato and potato led to reduced susceptibility to *B. cinerea*, which was associated with impediment of conidial germination and attachment as well as hyphal growth [29]. *Arabidopsis DMR1* (*Downy Mildew Resistance 1*) encodes a homoserine kinase that catalyzes the phosphorylation of homoserine to *O*-phospho-homoserine [30, 31]. *Arabidopsis dmr1* mutants were found to be resistant to *H. arabidopsidis*, the powdery mildew fungus *Oidium neolycopersici*, and two *Fusarium* pathogens [32–34]. In *dmr1* mutants, hyphal growth of *H. arabidopsidis* was arrested and underdeveloped haustoria were often surrounded by cell wall appositions containing callose [35]. The resistance observed in *dmr1* mutants was shown to be associated with accumulation of homoserine [32–35]. *Arabidopsis DMR6* (*Downy Mildew Resistance 6*) encodes a 2-oxoglutarate Fe(II)-dependent oxygenase [36]. *Arabidopsis dmr6* mutants showed reduced susceptibility to *H. arabidopsidis*, *P. syringae* pv. *tomato*, and *Phytophthora capsici* [34–36]. In the *dmr6* mutant, hyphal growth and haustoria development of *H. arabidopsidis* was observed, but the haustoria often had aberrant shapes and stayed immature [35]. Enhanced expression of defense-associated genes, including *PR1*, was suggested to contribute to the observed *dmr6*-mediated resistance [34, 36].

Very recently, studies have shown that CRISPR-generated mutants in these three individual genes led to resistance to various pathogens in different crops [37–40]. In previous studies, we showed that individual silencing of *StDND1*, *StDMR1*, and *StDMR6* increases potato resistance against *P. infestans*. To analyze how these *S*-gene-silenced plants prime defense responses to arrest colonization we monitored the infection process of *P. infestans* by microscopic and histological examination, and determined expression profiles of defense marker genes at early infection stages. Our results show that *P. infestans* infection in diverse *S*-gene-silenced potato plants is hindered at different stages.

## Results

### Increased resistance to multiple *P. infestans* isolates in *StDND1-*, *StDMR1-*, and *StDMR6-*silenced plants

**Figure 1:**
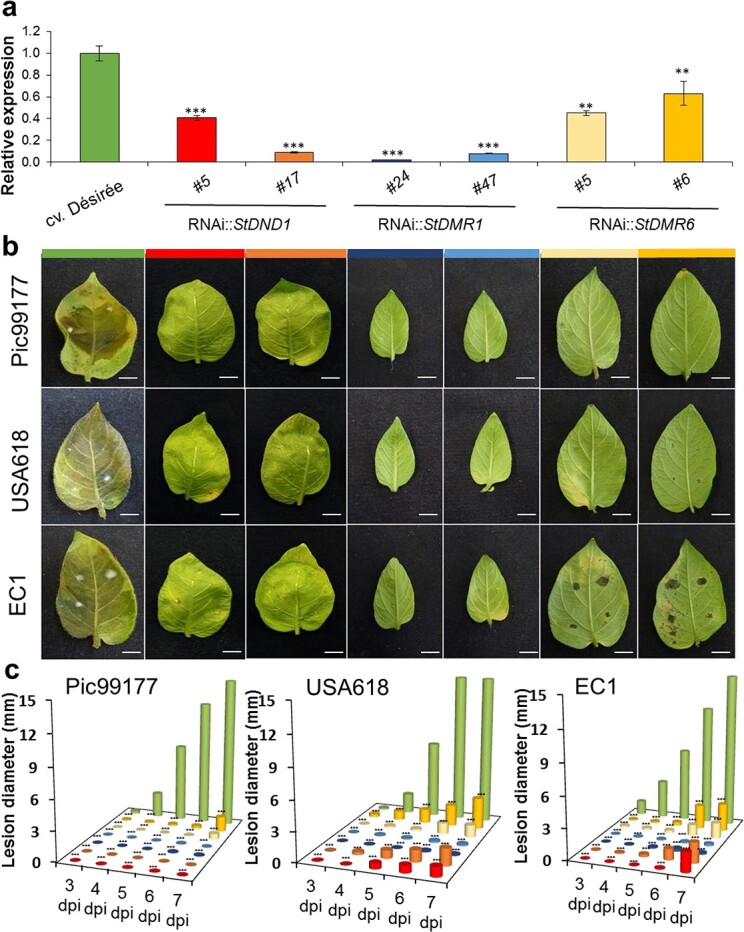
*StDND1*-, *StDMR1-*, and *StDMR6-*silenced potato lines show enhanced resistance to multiple *P. infestans* isolates. **a** Relative *S*-gene expression in leaves of potato cv. ‘Désirée’ and two independent potato transformants per RNAi genotype. **b** Disease symptoms on leaves of cv. ‘Désirée’ and *S*-gene-silenced lines after inoculation with the *P. infestans* isolates Pic99177, USA618, and EC1 at 7 dpi. Scale bars represent 10 mm. **c** Lesion development on leaves inoculated with *P. infestans* isolates Pic99177, USA618, and EC1. Three plants were used for each potato genotype, and each was spot-inoculated with 12 droplets of *P. infestans* inoculum. Asterisks indicate significant differences from the recipient cv. ‘Désirée’ (**P < .01; ***P < .001). Two independent experiments were performed with similar results.

**Figure 2:**
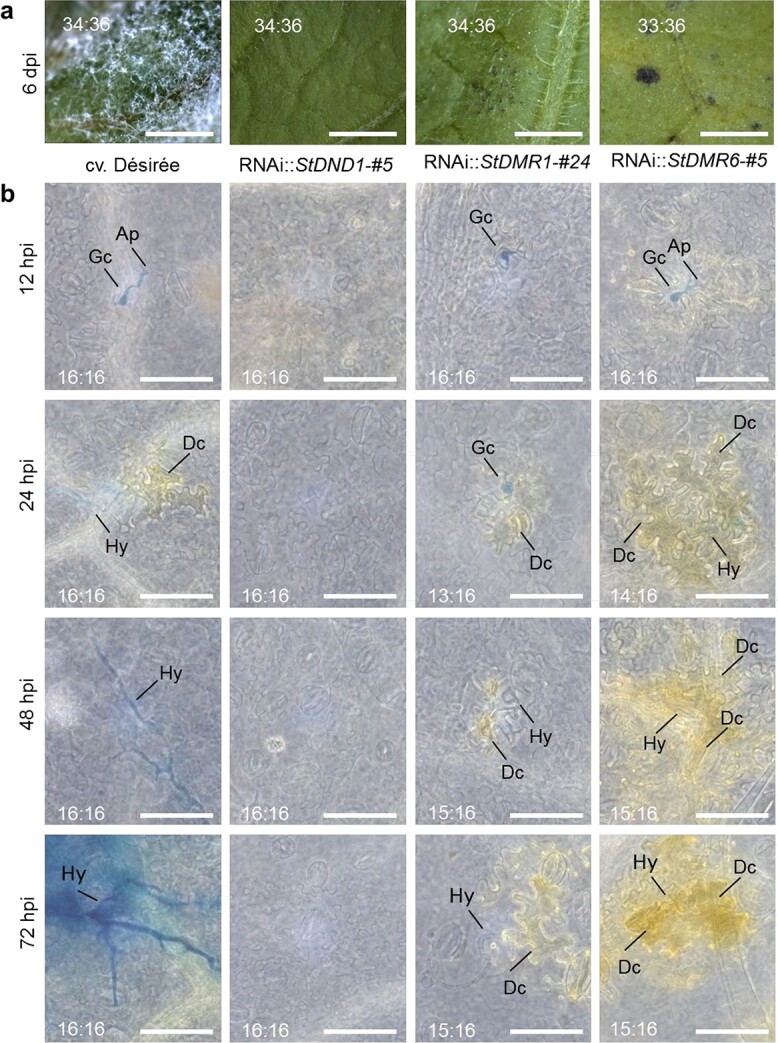
*S*-gene silencing in potato hampers *P. infestans* at different infection stages. **a** Disease symptoms on leaves of cv. ‘Désirée’ and *S*-gene silenced plants after inoculation with the *P. infestans* GUS-transformant EY6. Each image represents a single inoculation site at 6 dpi. The numbers in each image represent the ratio of inoculated sites with similar symptoms to the total number of inoculated sites in two independent experiments. Scale bars represent 5 mm. **b** Microscopic images of GUS-stained infection sites after inoculation with *P. infestans* EY6. The numbers in each image represent the ratio of inoculated sites with similar responses to the total number of inoculated sites in two independent experiments. Scale bars represent 100 μm. Ap, appressoria; Gc, germinated cyst; Hy, hypha; Dc, dead cell. Similar results were obtained with another set of RNAi lines (RNAi::*StDND1*-#17, RNAi::*StDMR1*-#47, RNAi::St*DMR6*-#6).

Previously, we demonstrated that silencing of *StDND1*, *StDMR1*, or *StDMR6* in potato results in enhanced resistance to *P. infestans* isolate Pic99189 [15], and additionally to three genetically diverse isolates, Pic99177, USA618, and EC1, upon silencing of *StDND1* [18]*.* In this study, we tested all three *S*-gene-silenced potato lines with multiple *P. infestans* isolates (Supplementary Table S1). The reduction in *S*-gene expression was quantified by qRT–PCR (Fig. 1a) and resistance was assessed in detached leaf assays by measuring lesion size at 3–7 days post-inoculation (dpi) in two independent experiments. All three tested isolates were able to establish infection on the susceptible control cv. ‘Désirée’ efficiently, resulting in sporulating lesions at 7 dpi (Fig. 1b). In contrast, lesion development on the *S-*gene silenced plants was largely hampered. *StDND1-* and *StDMR6***-**silenced plants showed no lesion growth upon inoculation with Pic99177 and, when inoculated with the aggressive isolates USA618 and EC1, the lesions were significantly smaller as compared with those on cv. ‘Désirée’. No lesion growth was observed on leaves of *StDMR1-*silenced plants inoculated with all three isolates (Fig. 1b and c). Dark necrotic spots surrounding the inoculation site were visible on *StDMR1-* and *StDMR6*-silenced plants at 6 dpi, whereas *StDND1-*silenced plants did not show such response (Fig. 2a). These results confirm that silencing *StDND1* leads to resistance to multiple *P. infestans* isolates [18] and show that the same holds true for the other two *S* genes, *StDMR1* and *StDMR6.* Further, as in our previous studies [15, 18] dwarfness was observed in *StDMR1-*silenced plants and autonecrosis in some of the *StDND1*-silenced plants.

### Hindered *P. infestans* infection in *StDND1-*, *StDMR1-*, and *StDMR6*-silenced potato plants

To follow the infection process in more detail, leaves were inoculated with zoospores of the transgenic *P. infestans* reporter strains 14-3-GFP and EY6 (GUS labeling) and microscopically monitored at different time points of infection [0–96 hours post-inoculation (hpi)]. To monitor strain EY6, a histological study was performed at seven time points (Supplementary Fig. S1). At the two earliest time points, i.e. 0 and 6 hpi, no zoospores were found on any of the genotypes, indicating that they were not attached to the leaf surface and thus were likely washed off during slide preparation. At 12 hpi, germinating cysts with primary appressoria were observed (Fig. 2; Supplementary Fig. S2). Leaf tissue below infection sites on *StDMR6*-silenced plants started turning yellow at 12 hpi (Fig. 2). From 24 hpi onwards, intracellular hyphae were observed in leaves of cv. ‘Désirée’, whereas on *StDMR1*- and *StDMR6*-silenced plants cysts with a short germination tube and intercellular hyphae were found (Fig. 2; Supplementary Fig. S2). Exceptions were *StDND1*-silenced plants, on which no *P. infestans* was observed at any time point (Fig. 2).

Growth and proliferation of *P. infestans* were analyzed in detail by monitoring isolate 14-3-GFP at eight time points (Supplementary Fig. S1). Three hours after inoculation with *P. infestans* cysts, germination was observed on all plants, except on *StDND1*-silenced lines (Supplementary Fig. S3a). On cv. ‘Désirée’ plants, hyphal elongation started at 16 hpi. Extensively branched hyphae with collapsed cells underneath became evident at 48 hpi (Supplementary Fig. S3b). Mycelium developed sporangiophores from 72 hpi onwards, releasing numerous sporangia at 96 hpi. On *StDMR1-* and *StDMR6-*silenced plants, the development of *P. infestans* was similar to that observed on cv. ‘Désirée’ at 6 and 16 hpi (Supplementary Fig. S3a). However, at later time points hyphal elongation was arrested (Supplementary Fig. S3b). This was associated with local cell death at the infection sites that became apparent at 16 hpi (Supplementary Fig. S3a). On *StDND1*-silenced plants, only germinated cysts with short germ tubes were found (Supplementary Fig. S3).

**Figure 3:**
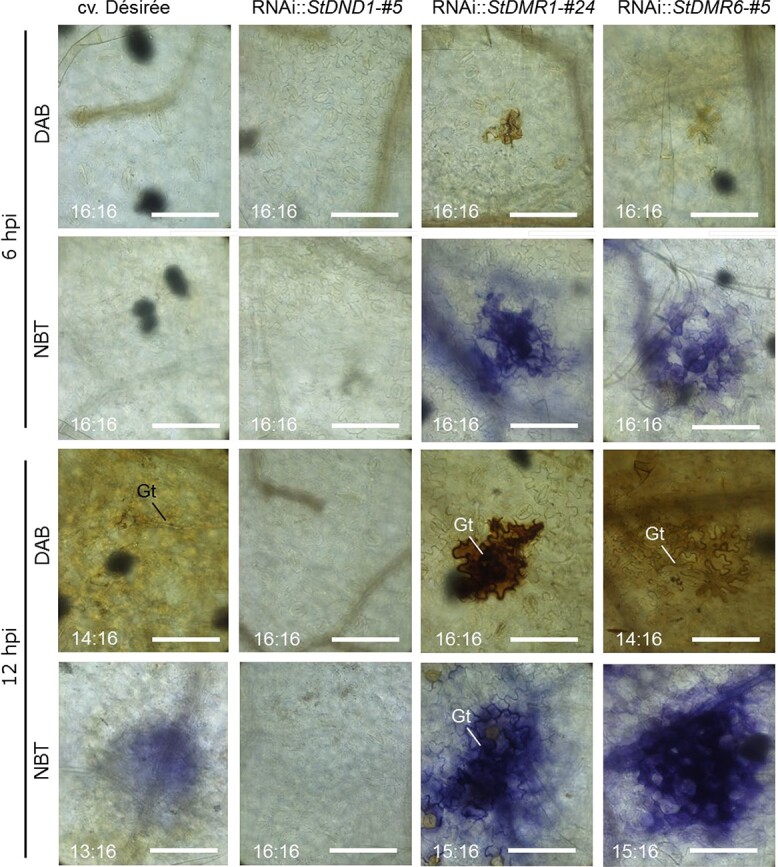
*S*-gene silencing enhances ROS accumulation at early time points of infection. Microscopic images of potato leaves stained with DAB (H_2_O_2_) and NBT (O_2_^−^) at 6 and 12 hpi with *P. infestans* EY6. Each image represents a single inoculation site. The numbers in each image represent the ratio of inoculated sites with similar responses to the total number of inoculated sites in two independent experiments. Scale bars represent 100 μm. Gt, germ tube. Similar results were obtained with another set of RNAi lines (RNAi::*StDND1*-#17; RNAi::*StDMR1*-#47; RNAi::St*DMR6*-#6).

### *StDMR1*- and *StDMR6-*silenced plants display early ROS accumulation upon *P. infestans* inoculation

To further determine the different interaction stages, we investigated ROS accumulation in leaves inoculated with *P. infestans* EY6 (Supplementary Fig. S1), using 3,3-diaminobenzidine (DAB) and nitroblue tetrazolium (NBT) staining to monitor generation of hydrogen peroxide (H_2_O_2_) and superoxide anions (O_2_^−^), respectively. On the *StDMR1-* and *StDMR6-*silenced plants, H_2_O_2_ and O_2_^−^ accumulation was observed at the inoculation sites starting from 6 hpi (Fig. 3). On cv. ‘Désirée’ plants, O_2_^−^ and H_2_O_2_ production became evident starting from 12 and 24 hpi, respectively (Fig. 3; Supplementary Fig. S4). Compared with cv. ‘Désirée’ plants, where ROS accumulation expanded beyond the inoculation sites at 48 hpi, it was limited to cells at the original inoculation site until 72 hpi on *StDMR1-*silenced plants and until 48 hpi on *StDMR6-*silenced plants (Fig. S4). In inoculated *StDND1-*silenced plants there was no accumulation of H_2_O_2_ or O_2_^−^ (Fig. 3; Supplementary Fig. S4).

### Changes in defense-related gene expression in *S*-gene-silenced plants

**Figure 4:**
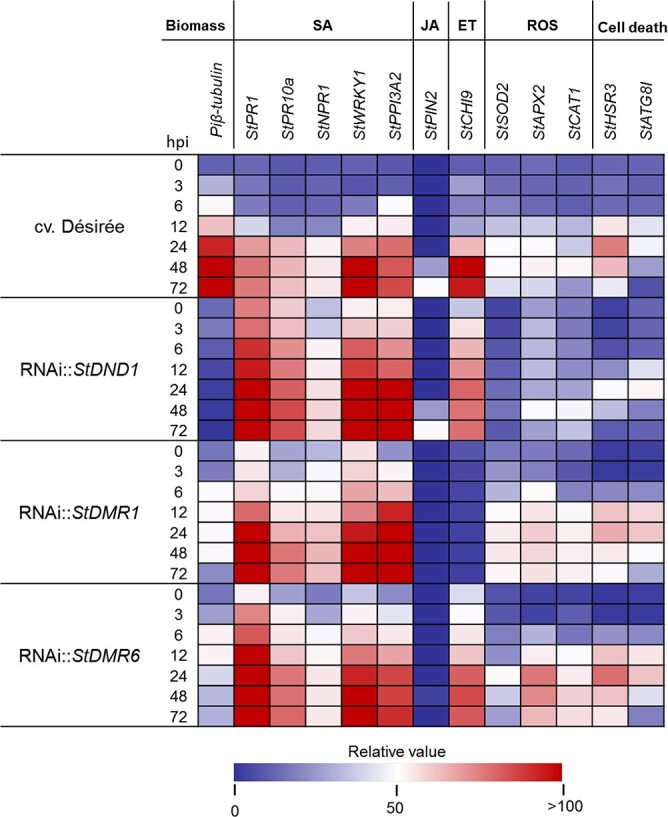
Expression profiles of potato defense marker genes in *S-*gene-silenced potato lines during *P. infestans* infection. Relative expression of defense genes in *StDND1-*, *StDMR1-*, and *StDMR6-*silenced potato lines and its recipient cv. ‘Désirée’ upon inoculation with *P. infestans* EY6. Values are average gene expression levels measured in three biological replicates, each consisting of three technical repeats (Supplementary Figs S5–S10). Expression of potato *EF1a* (*StEF1a*) was used as endogenous control, and values were calculated relative to expression levels at 0 hpi in cv. ‘Désirée’. Pathogen biomass in leaves was quantified relative to *Piβ-tubulin* expression.

To confirm the microscopic observations and validate our sampling procedure, we quantified pathogen biomass by qRT–PCR at various times after inoculation with strain EY6 (Fig. 4; Supplementary Fig. and Fig S5). In cv. ‘Désirée’ plants there was a steady increase in *P. infestans* biomass starting from 3 hpi, reaching a plateau at 24 hpi. In contrast, *P. infestans* biomass was low in leaves of RNAi plants, confirming increased resistance to *P. infestans* acquired by silencing of *StDND1*, *StDMR1*, and *StDMR6* (Fig. 4; Supplementary Fig. S5).

To determine which signaling pathways play a role in the acquired resistance, we examined the expression of several defense-related marker genes by qRT–PCR (Fig. 4 and; Supplementary Figs S6 and S8). These included *StPR1*, *StPR10a*, *StNPR1*, *StWRKY1*, and *StPP13A2* for the salicylic acid (SA) pathway, *StPIN2* for the jasmonate (JA) pathway, and *StCHI9* for the ethylene (ET) pathway [41–43]. Induction of expression of SA marker genes was observed in cv. ‘Désirée’ starting from 12 hpi, in contrast to earlier induction in the *S*-gene-silenced lines. The JA-responsive marker *StPIN2* was slightly upregulated in cv. ‘Désirée’ and *StDND1-*silenced plants at 48 and 72 hpi while no or hardly any expression was observed in *StDMR1-* and *StDMR6-*silenced plants. The ET marker gene *StCHI9* showed a clear increase in expression in cv. ‘Désirée’ from 24 hpi onwards. An earlier, but weaker, induction of *StCHI9* expression was detected in *StDND1-* and *StDMR6-*silenced plants. These data suggest that in *StDMR1-*silenced plants only the SA-mediated signaling pathway contributes to increased resistance to *P. infestans*, while in *StDND1-* and *StDMR6-*silenced plants ET-mediated signaling may contribute as well.

To further validate the oxidative burst and cell death responses observed in inoculated *StDMR1-* and *StDMR6-*silenced plants, we also evaluated the expression of marker genes associated with these events (Fig. 4; Supplementary Figs S9 and S10). We included three genes encoding ROS-scavenging or ROS-generating enzymes, i.e. superoxide dismutase (*StSOD2*), ascorbate peroxidase (*StAPX2*), and catalase (*StCAT1*), and two cell death-related genes, *StHSR3* and *StATG8I*. Compared with cv. ‘Désirée’, slightly higher induction of expression of these genes was observed in *StDMR1-* and *StDMR6-*silenced plants from 6 hpi onwards. In contrast, expression of these marker genes was hardly detectable in *StDND1-*silenced plants (Fig. 4), and thus these expression patterns match the ROS accumulation observed in the respective RNAi plants (Figs 2 and 3; Supplementary Fig. S3).

## Discussion

This study shows that broad-spectrum resistance to *P. infestans* resistance can be efficiently achieved by silencing of *StDND1*, *StDMR1*, or *StDMR6* in potato [5, 18]. This is in line with several recent studies that realized resistance to a number of pathogens by CRISPR editing these three individual genes in different crops [37–40], demonstrating the importance of *S*-genes in breeding crops with improved disease resistance.

**Figure 5:**
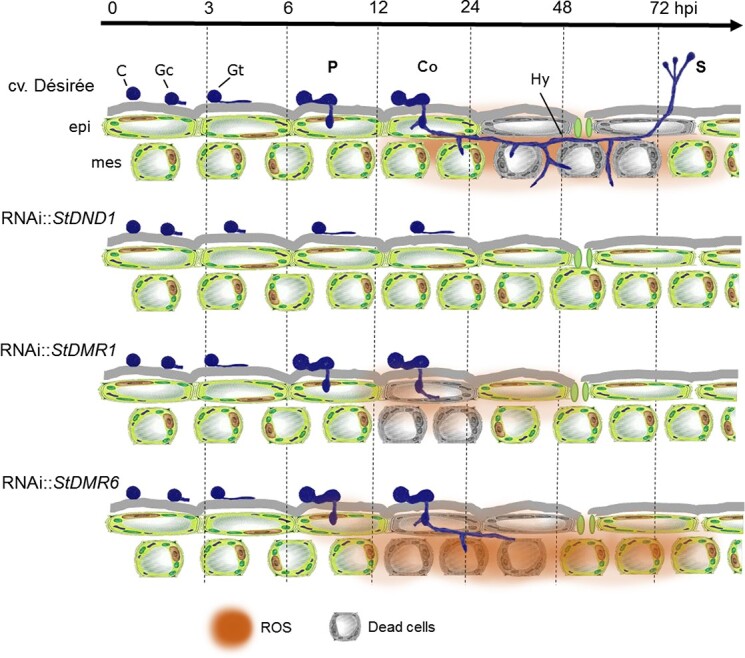
*S*-gene silencing in potato arrests colonization by *P. infestans* at different stages of early infection. Model depicting differences in *P. infestans* colonization on leaves of cv. ‘Désirée’ (susceptible; top panel) and *S*-gene-silenced potato lines (resistant; lower panels). On *StDND1*-silenced lines, *P. infestans* is arrested prior to host penetration of epidermal cells (P). *StDMR1-*silenced plants display strong epidermal cell death at sites of penetration, whereas *StDMR6-*silenced plants arrest *P. infestans* colonization (Co) by enhanced cell death in the mesophyll layer. *P. infestans* is able sporulate (S) to complete its lifecycle on cv. ‘Désirée’ within 72 hpi. C, cyst; Gc, germinated cyst; Gt, germ tube formation.

Compared with our previous studies and those reported in the literature, this study gathered insight into the infection process of *P. infestans* on *S*-gene*-*silenced potato plants by making use of GUS(β-glucuronidase)- and GFP(green fluorescent protein)-labeled *P. infestans* strains. On *StDND1-*silenced plants, penetration of *P. infestans* was hampered and growth was arrested after cyst germination. On *StDMR1-* and *StDMR6-*silenced plants, growth of *P. infestans* was hindered after germ tube emergence, which was associated with cell death and ROS accumulation at the inoculation sites (Fig. 5).

For monitoring the activity of defense-related signaling pathways we made use of marker genes. Resistance mediated by silencing *StDMR1* was found to be correlated with the early induction of SA-mediated signaling. Although delayed germination and no penetration were observed on *StDND1-*silenced plants, it is intriguing to notice that, in both *StDND1*- and *StDMR6*-silenced plants, the increased resistance was associated with an early onset of SA- and ET-mediated signaling pathways. It thus seems that *P. infestans* germ tubes release signals that potentially induce defense signaling. The association between the microscopic observations and defense-related gene expression in the *S*-gene-silenced plants may offer leads for further exploration of the mechanisms underlying enhanced resistance to *P. infestans*.

The *dnd1* mutant of *Arabidopsis* exhibits enhanced resistance to avirulent isolates of *P. syringae*, which is not associated with a hypersensitive response [27, 28]. In agreement with this, our study demonstrated that a hypersensitive response does not contribute to the acquired resistance to *P. infestans* by silencing the potato *DND1* ortholog. Instead, all (germinated) cysts of the GUS-expressing line EY6 were washed off during slide preparation, suggesting that cyst attachment to the plant surface of *StDND1***-**silenced plants is blocked. This lack of attachment was also observed when the *StDND1***-**silenced plants were inoculated with *B. cinerea* [29]. It might be that the leaf surface of *StDND1***-**silenced plants is chemically and/or physically altered. However, we have no direct evidence to prove the lack of cyst attachment. When no washing steps were included in preparing samples with the GFP-labeled isolate, cysts germinated and produced short germ tubes (Supplementary Fig. S3). Further, similar expression profiles were found for both the *StDND1-* and *StDMR6-*silenced plants. These observations may indicate that cysts do attach to the leaf surface of *StDND1*-silenced plants but are unsuccessful in penetration. A recent study showed that *Phytophthora* species, including *P. infestans*, use a slicing mechanism to invade their hosts. The tip of the germ tube transforms into a knife-like structure that cuts through the plant surface at an oblique angle [45]. Using this strategy, *Phytophthora* is able to penetrate without brute force and with minimal consumption of energy. This study further implied that according to the laws of mechanics *Phytophthora* is unable to penetrate the plant without first attaching itself tightly to the leaf surface. Taking this into consideration, we speculate that the leaf surface of *StDND1*-silenced plants is physically altered, and as a result the pathogen cannot attach and penetrate. The *DND1* gene encodes a protein that is a member of the CNGC family but as yet there is no indication of direct involvement of CNGC family members in cell wall biogenesis or cuticle formation. CNGCs play roles in conducting Ca^2+^ into plant cells and are involved in various physiological processes [27]. The *Arabidopsis dnd1* mutant displays elevated SA levels, which are likely required for its resistance to a broad range of pathogens [23]. In the *StDND1***-**silenced potato plants, we observed a constitutively elevated expression of SA marker genes indicative of elevated SA levels (Fig. 5; Supplementary Fig. S6, [18]). The elevated levels of *PR1* expression in *StDND1-*silenced plants may lead to increased content of PR1 protein, which has been shown to have inhibitory activity against *P. infestans* [44]. Further, the ET marker gene *StCHI9* was induced upon *P. infestans* infection in the *StDND1***-**silenced potato plants (Fig. 5: Supplementary Fig. S8). This suggests that the increased resistance to *P. infestans* found in the *StDND1***-**silenced potato plants relies on both SA- and ET-mediated signaling pathways.

In *Arabidopsis dmr1* mutants, pathogen resistance without visible hypersensitive response [35] to *H. arabidopsidis*, *O. neolycopersici*, *Fusarium culmorum*, and *Fusarium graminearum* was found to be associated with homoserine accumulation [32, 33, 35]. Our results show clear cell death of single epidermal cells of *StDMR1-*silenced potato plants at the site of *P. infestans* inoculation. This ensures pathogen arrest at a very early infection stage, when cysts start to germinate. *P. infestans* is a hemibiotroph that requires an initial biotrophic phase in which nutrients from living host cells are acquired via intracellular haustoria [46]. A rapid host cell death observed in *StDMR1*-silenced plants thus strongly hinders this crucial biotrophic growth phase (Fig. 3). In contrast to *Arabidopsis dmr1* mutants [31], induction of SA marker gene expression and ROS accumulation were detected in the *StDMR1***-**silenced potato plants.

*Arabidopsis dmr6* mutants were shown to have a reduced susceptibility to *H. arabidopsidis*, *P. syringae* (only at adult stage), and *P. capsici* [34–36]. In the *dmr6* mutant, haustorium formation of *H. arabidopsidis* was found to be severely affected; haustoria had aberrant shapes and stayed immature [35]. Here we found that death of multiple cells occurred on *StDMR6-*silenced plants around 16 hpi after *P. infestans* inoculation, which was associated with induced expression of SA and ET marker genes. *DMR6* encodes a 2-oxoglutarate Fe(II)-dependent oxygenase, suggesting that the observed resistance is caused by the accumulation of a toxic DMR6 substrate or the absence of a DMR6 metabolic product required for pathogen growth [34]. Later, it was shown this was accompanied by elevated SA levels, and that DMR6 functions in a feedback mechanism to tightly control the SA level [36].

Potato breeding for late blight resistance has so far relied on the introgression of dominant resistance (*R*) genes from crossable relatives [1]. However, *R-*gene-mediated resistance is not always durable; *P. infestans* can easily avoid recognition by mutating or deleting RXLR effector genes so that NLRs encoded by dominant *R-*genes are not activated to mount effective defense [2]. Thus, we previously proposed an alternative/additive strategy to enhance late blight resistance by impairing plant *S*-genes that are required by *P. infestans* for successful host colonization [47]. In potato, several of these *S*-genes have been identified, including genes encoding a KRBP1 protein [K-homology (KH) RNA-binding protein], three isoforms of the PP1c protein (phosphatase PP1 catalytic subunits) and an NRL1 protein [non-phototrophic hypocotyl 3/root phototropism 2 (NPH3/RPT2)-like protein]. These three host proteins are targeted by the *P. infestans* RXLR effectors Pi04314, Pi04089, and Pi02860 to promote infection [48–50]. Some *S-*genes function as negative regulators of plant defense, which are often upregulated by pathogens to suppress plant defense [19]. Therefore, silencing of *S-*gene expression by RNAi can avoid the suppression of plant defense by pathogens. RNAi is especially powerful in polyploid crops like potato since it results in a dominant inherited resistance [15]. Recent advances in genome editing, including CRISPR/Cas9 technology, make *S-*gene editing a promising strategy for resistance breeding, even in tetraploid potato and other polyploid crops [51].

The three *S*-genes studied here were not identified by screening potato for susceptibility factors towards late blight disease. Instead, they were pinpointed as potential candidates because they are orthologs of *S*-genes identified in *Arabidopsis*. Our results showed that these orthologs can also be functionally conserved across plant species. For example, downregulation of *DND1* expression in potato and tomato led to resistance to powdery mildew, late blight, and grey mold disease [18, 29]. Downregulation of *DMR1* expression in tomato and pepper was shown to enhance resistance to powdery mildew and *P. capsici*, respectively [33, 52]. Similarly, tomato *dmr6* mutants generated by CRISPR/Cas9 were found to be resistant to *P. capsici*, as well as *P. syringae* pv. *tomato* [53]. These results, including those presented here, show that impairment of orthologous *S*-genes in diverse crop species potentially leads to broad-spectrum resistance to multiple/diverse pathogens. However, further studies are needed to fully unravel the molecular basis of resistance mediated by loss-of-function mutation of *S*-genes in different plant species.

## Materials and methods

### Pathogen growth and inoculum preparation

The *P. infestans* isolates used in this study (Supplementary Table S1) are genetically diverse isolates, including Pic99177, USA618, EC1, EY6 (88069), and 14–3-GFP (H30P02). They were cultured on rye sucrose agar medium in the dark at 15°C for 10–14 days. Agar plates fully covered with sporulating mycelium were flooded with cold water (4°C). Sporangiospore suspensions were harvested and incubated at 4°C for 1–2 hours to induce zoospore release. Zoospores were isolated by filtration through 15-μm nylon mesh. Experimental procedures are visualized in Supplementary Fig. S1.

### Plant growth conditions and infection assays

Three-week-old *in vitro*-propagated potato plantlets were transferred to soil and grown in a greenhouse at 25 ± 2°C with 75% relative humidity and a 16:8 hour (light/dark) photoperiod. *S*-gene-silenced lines RNAi::*StDND1*-#5, −#17; RNAi::*StDMR6*-#5, −#6; and RNAi::*StDMR1*-#24, −#47 were previously described by Sun *et al*. [15], and are derivatives of potato cv. ‘Désirée’ (R0), which was used as non-transgenic control throughout the experiments. Mature potato plants with fully developed composite leaves were used for detached leaf assays as described by Vleeshouwers *et al*. [54]. Harvested leaves were arranged in floral foam and placed in plastic boxes with a transparent lid. Abaxial leaf surfaces were inoculated with 10-μl droplets of inoculum (2.5 × 10^4^ zoospores/ml), and subsequently incubated at 18°C with a 16/8 hour photoperiod. Lesion diameters were measured 3–7 dpi using a caliper with a digital display (DIGI-MET^®^, Helios Preisser, Germany). Disease assays were repeated twice, each consisting of >30 inoculation spots per potato line (Supplementary Fig. S1).

### Microscopy and histochemical staining

Host penetration and infection by *P. infestans* was assessed by imaging leaf disks (diameter 10 mm) punched out from spot-inoculated leaf areas. Bright-field images were taken with a Zeiss Stemi 508 stereomicroscope equipped with a digital camera. Accumulation of H_2_O_2_ and O_2_^−^ was visualized by DAB and NBT staining, respectively [55]. To optimize staining, leaf disks were kept overnight in staining solutions. Tissue clearing was performed by boiling the leaf disks in 96% ethanol. The leaf disks were subsequently kept in 70% ethanol until examination. Histochemical GUS staining was performed as described by Sun *et al*. [29]. Fluorescence microscopy was performed with a Nikon 90i epifluorescence microscope equipped with a GFP-LP filter and a digital Nikon DS-5MC camera. For each time point, at least eight leaf disks per potato genotype were observed. Each bioassay was performed at least twice, with the exception of the histochemical GUS assay. Pictures show single infection sites.

### Nucleic acid extraction and gene expression analyses

Leaf samples were flash-frozen, ground in liquid nitrogen, and stored at −80°C. Total RNA was extracted using a MagMAX-96 Total RNA Isolation Kit (Ambion) and treated with RNAse-free DNAse (Qiagen). RNA concentrations were measured with an Isogen Nanodrop Spectrophotometer ND-1000. Synthesis of cDNA was performed on 1 μg of total RNA using an iScript cDNA Synthesis Kit (Bio-Rad). Quantitative RT–PCR was performed on a C1000™ Thermal Cycler PCR system (Bio-Rad) using iQ SYBR Green Supermix (Bio-Rad). Gene-specific primers were designed with the online tool Primer3 (Supplementary Table S2). Expression levels of marker genes were quantified with the 2^-ΔΔCt^ method using *StEF1α* and *Piβ-tubulin* expression for normalization [15]. Quantitative RT–PCR assays were performed on three independent biological samples, each consisting of three technical replicates.

## Statistical analysis

Data presented are means of at least three biological replicates with error bars indicating standard deviation. Statistical analyses for detached leaf assays and gene expression were conducted using one-tailed *t*-tests and Duncan’s multiple range test performed in SPSS, respectively.

## Acknowledgements

We thank Dirk Jan Huigen and Gerda van Engelenhoven for maintaining the potato plant material, Ageeth van Tuinen and Marjon Arens for their assistance in disease assays. This work was financially supported by the University Fund Wageningen, TKI Uitgangsmaterialen (EZ-2012-07), the National Science Foundation of China (31801420), and the Scientific and Technological Project of Henan Province (202102110187).

## Author contributions

K.S., K.B., E.J., F.G., and Y.B. designed this research. K.S., D.S., and K.B. collected and analyzed the data. K.S., K.B., and Y.B. wrote the manuscript. F.G., E.J., and R.G.F.V. edited the manuscript.

## Data availability

All datasets supporting the conclusions of this article are included within the article (and its Supplementary material files).

## Conflict of interest

The authors declare that they have no competing interests.

## Supplementary data

Supplementary data is available at *Horticulture Research* online.

## Supplementary Material

Web_Material_uhab058
