# Supplementary material for: Silencing susceptibility genes in potato hinders primary infection with Phytophthora infestans at different stages
Source: Hortic Res. 2022 Jan 19;9:uhab058. doi: 10.1093/hr/uhab058 (PMC8968627; doi:10.1093/hr/uhab058)
Supplement: Web_Material_uhab058 [file web_material_uhab058.zip › Supplementary material.docx]

**Supplementary material**

**Table S1. *Phytophthora infestans* isolates used in this study**

|  | **Race** | **Aggressiveness** ^a^ | **MT** | **Reference** |
| --- | --- | --- | --- | --- |
| **Isolate** | | | | |
| Pic99177 | 1.2.3.4.7.9.11 | moderately aggressive | A2 | ^1^ |
| EC1 | 1.3.4.7.10.11 | aggressive | n.d. | ^2^ |
| USA618 | 1.2.3.6.7.10.11 | aggressive | A2 | ^3^ |
| **Transformant** (background) | | | | |
| EY6 (88069) | 1.3.4.7 | n.d. | A1 | ^4^ |
| 14-3-GFP (H30P02) | 3a.7.10 | n.d. | n.d. | ^5^ |

^a^ as determined by Champouret et al. (2009) on cv. Désirée. MT, mating type; n.d., not determined.

**References**

1 Flier, W. G. *et al.* *Phytophthora ipomoeae* sp. nov., a new homothallic species causing leaf blight on *Ipomoea longipedunculata* in the Toluca Valley of central Mexico. *Mycological Research* **106**, 848-856 (2002).

2 Armstrong, M. R., Whisson, S. C., Pritchard, L., Bos, J. I. B. & Birch, P. R. J. An ancestral oomycete locus contains late blight avirulence gene *Avr3a*, encoding a protein that is recognized in the host cytoplasm. *Proc Natl Acad Sci U S A* **102**, 7766-7771 (2005).

3 Fabritius, A. L. & Judelson, H. S. Mating-type loci segregate aberrantly *in Phytophthora infestans* but normally in *Phytophthora parasitica*: implications for models of mating-type determination. *Current Genetics* **32**, 60-65 (1997).

4 Van West, P., de Jong, A. J., Judelson, H. S., Emons, A. M. C. & Govers, F. The *ipiO* gene of *Phytophthora infestans* is highly expressed in invading hyphae during infection. *Fungal Genetics and Biology* **23**, 126-138 (1998).

5 Bouwmeester, K. *et al.* The Arabidopsis lectin receptor kinase LecRK-I.9 enhances resistance to *Phytophthora infestans* in Solanaceous plants. *Plant Biotechnology Journal* **12**, 10-16 (2014)

**Table S2. Q-RT-PCR primers used in this study.**

| **Primer name** | **Sequence (5'-3')** | **Used for** |
| --- | --- | --- |
| Fw-*Piβ-tubulin* | GGTCGTGGAGCCCTATAACG | Normalisation |
| Rv-*Piβ-tubulin* | GTCACCATAAGTGGGGGTGG |  |
| Fw-*StEF1a* | ATTGGAAACGGATATGCTCCA | Normalisation |
| Rv-*StEF1a* | TCCTTACCTGAACGCCTGTCA |  |
| Fw-*StPR1* | TGGTGATTTCACGGGGAGGG | Determination relative transcript levels |
| Rv-*StPR1* | CGAACTGAGTTGCGCCAGAC |  |
| Fw-*StPR10a* | TTTGAAGCTCATGGAAATGGAGG |  |
| Rv-*StPR10a* | AAGTTCCATGCCTTGTTTTTGGC |  |
| Fw-*StNPR1* | AGAGAGATGCGTCGCAACC |  |
| Rv-*StNPR1* | CAGTTTCATGGGCAGATCATCG |  |
| Fw-*StWRKY1* | GAAGAATAAAGCCGGGTTCTTGG |  |
| Rv-*StWRKY1* | CTTACACGATTTGATCACCTCATCC |  |
| Fw-*StPPI3A2* | GAGAAGGAAAATACACTTATAGCTG |  |
| Rv-*StPPI3A2* | GAACTCGATTACAACTAAAATCAGC |  |
| Fw-*StPIN2* | TTTGGTCCCTCGTCTCATTC |  |
| Rv-*StPIN2* | TGCCATGGAAAATGTAGCAA |  |
| Fw-*StCHI9* | GTGCTGCGAAAGGCTTCTAT |  |
| Rv-*StCHI9* | GTTGCCCATCCACCAGTAGT |  |
| Fw-*StSOD1* | CCTCTCACTGGTTCACAATCCA |  |
| Rv-*StSOD1* | TGGCCTCCAGGGTTAATTGCT |  |
| Fw-*StAPX1* | CCATTTGGAACAATCAGGCACCC |  |
| Rv-*StAPX1* | TGAAGTTACGGGAGGCCCCG |  |
| Fw-*StCAT* | GCCATGAACATGACACATCG |  |
| Rv-*StCAT* | CCCTGCCTGTTTGAAGTTGT |  |
| Fw-*StHSR3* | AACAAGGATGACCCGAGTTG |  |
| Rv-*StHSR3* | GTACTAGCGCTCCGTGAACC |  |
| Fw-*StATG8I* | CTGACCTCCCTGAGATGGAA |  |
| Rv-*StATG8I* | TTGTTGTTTGAGGCAAGGTG |  |

**
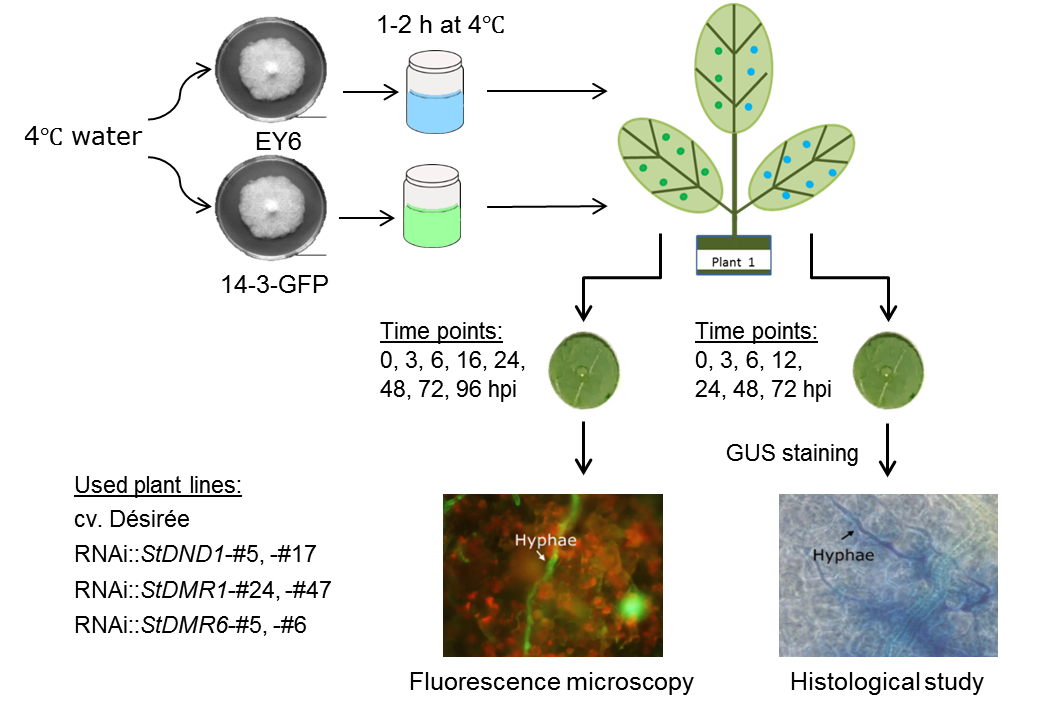
**

**Fig. S1. Experimental overview of histological and Q-RT-PCR assays**

For histological analyses, four compound leaves of each potato line (i.e. cv. Désirée and all *StDND1-*, *StDMR1-* and *StDMR6*-silenced lines) were spot-inoculated with zoospores of *P. infestans* isolate EY6 and 14-3-GFP, respectively. For Q-RT-PCR, another three leaves per potato line were inoculated with *P. infestans* EY6. Inoculation with MQ water was used as control. At each time point, four leaf discs were collected per leaf for the microscopic observations and six leaf discs were collected per leaf for Q-RT-PCR assays.

**
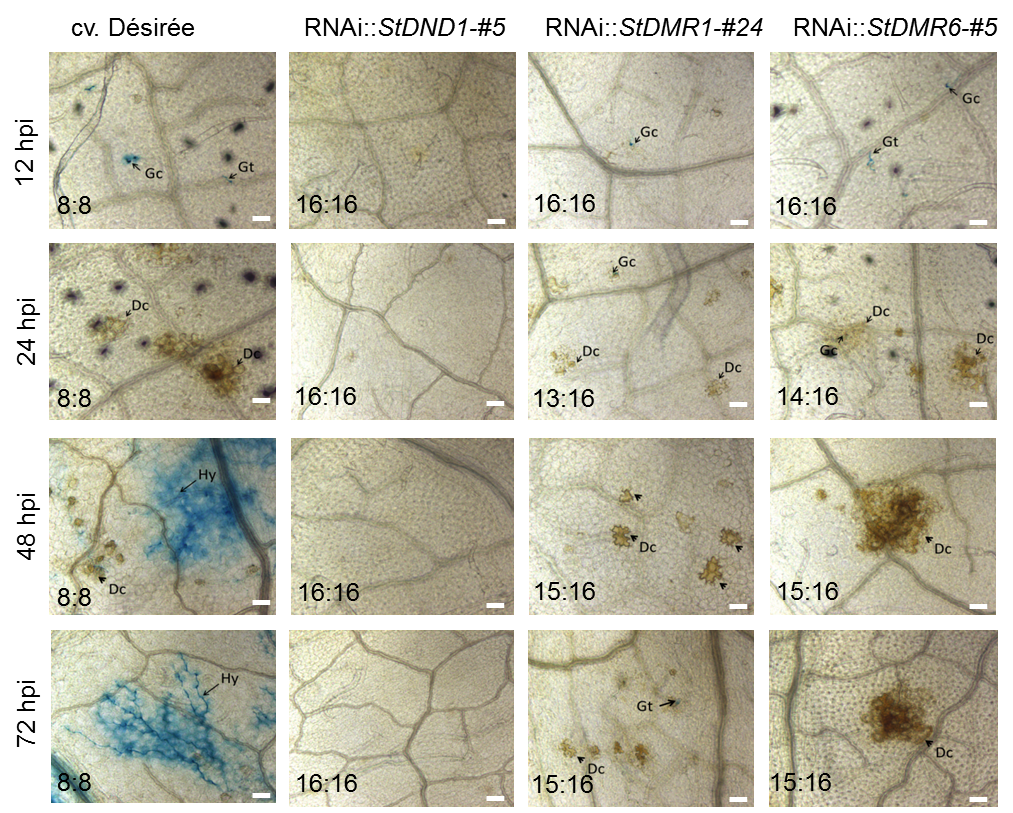
**

**Fig. S2. GUS-stained leaf discs of *S*-gene silenced potato lines with *P. infestans* EY6 at 12-72 hpi.** Each image represents a single inoculation site. The numbers in each image represent the ratio of inoculated sites with similar responses and the total inoculated sites. Scale bars represent 100 μm. Dead cells (Dc); germinated cyst (Gc); germ tube (Gt); hyphae (Hy).


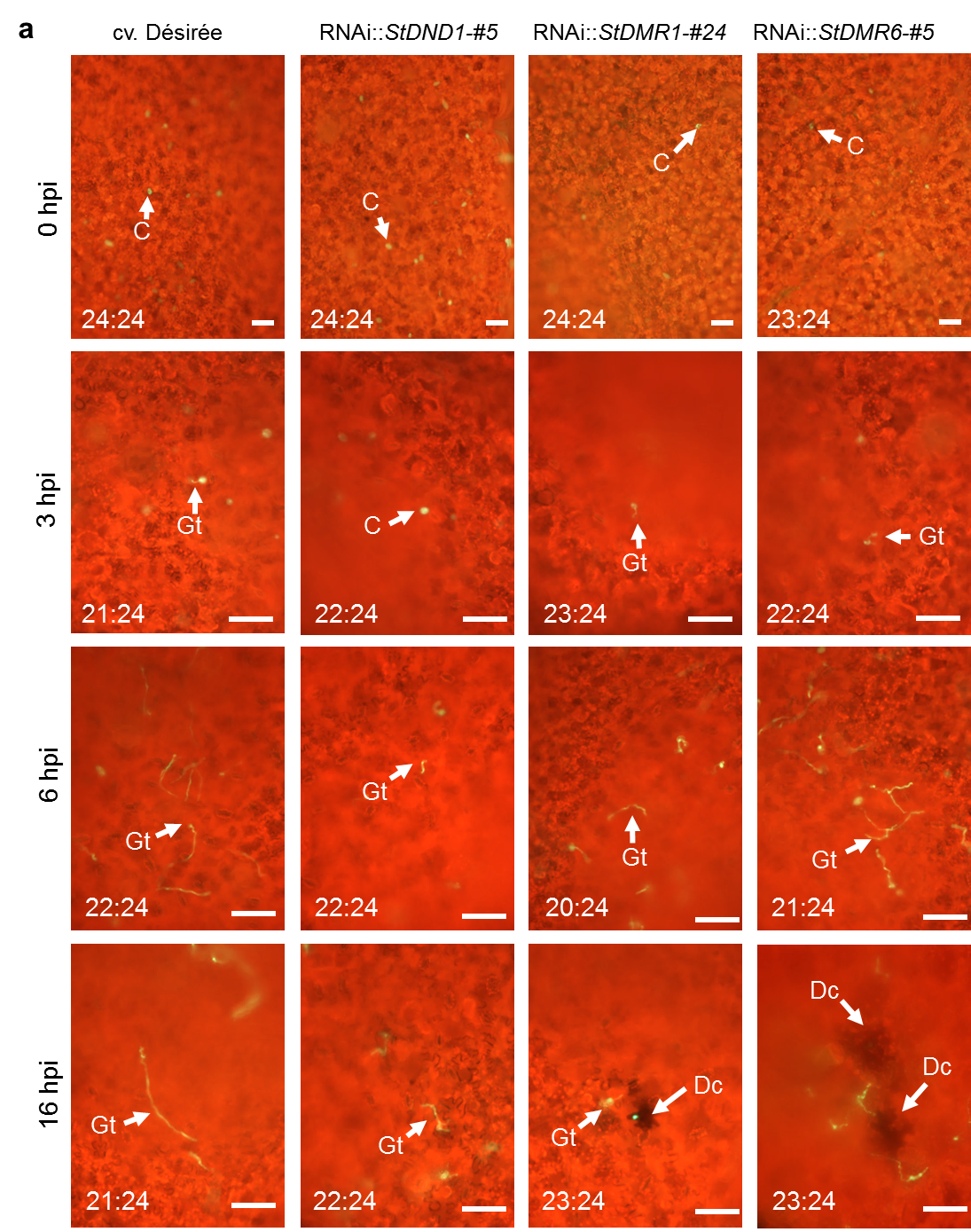


**
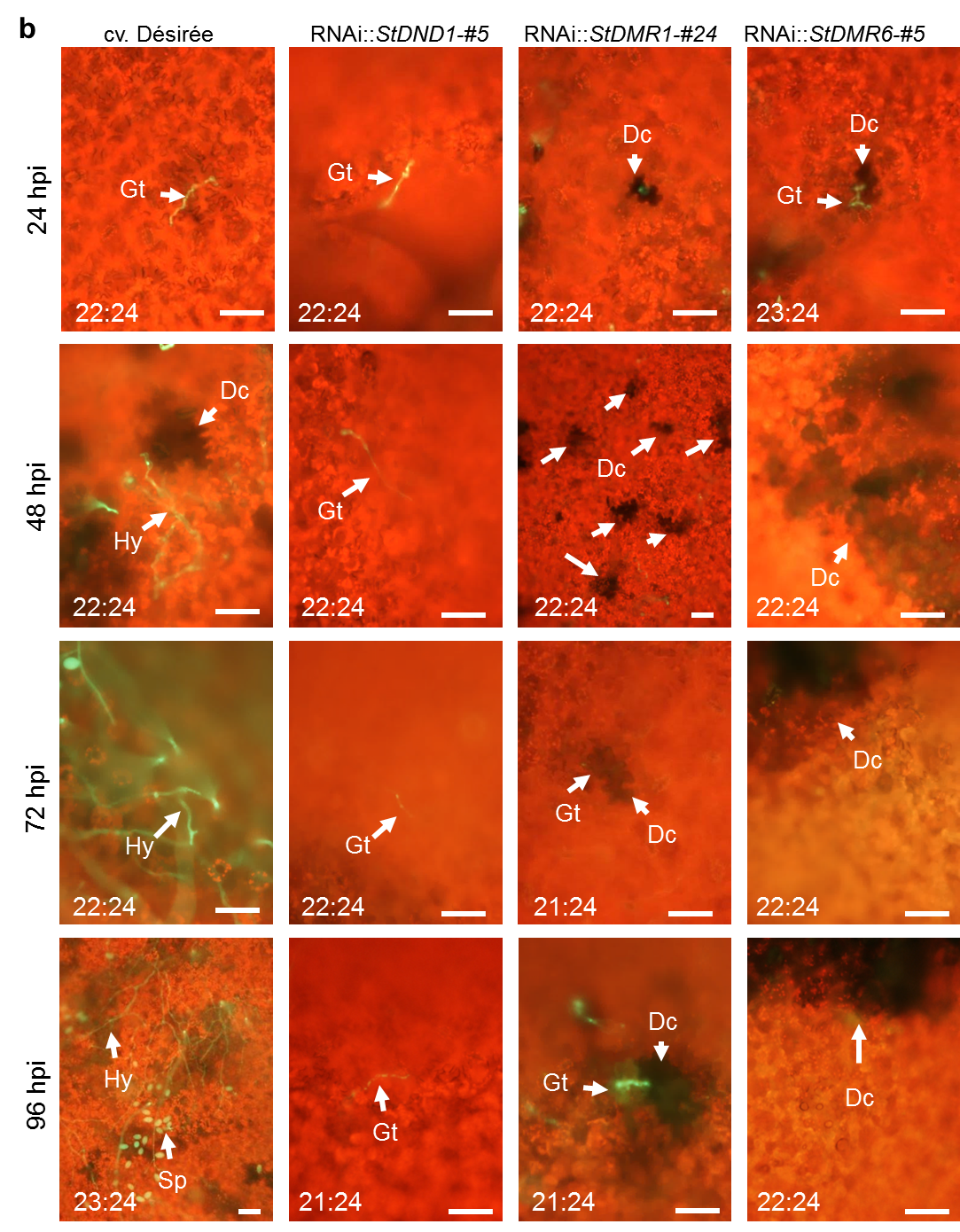
**

**Fig. S3. Infection of *P. infestans* 14-3-GFP** **on *S*-gene silenced potato plants.** Fluorescence microscopic images of potato leaves at 0-16 hpi (**a**), and 24-96 hpi (**b**) with *P. infestans* 14-3-GFP. Each image represents a single inoculation site. The numbers in each image represent the ratio of inoculated sites with similar responses and the total inoculated sites. Scale bars represent 100 μm. Cysts (C), dead cells (Dc), germ tube (Gt), hyphae (Hy), sporangiophore (Sp).

**
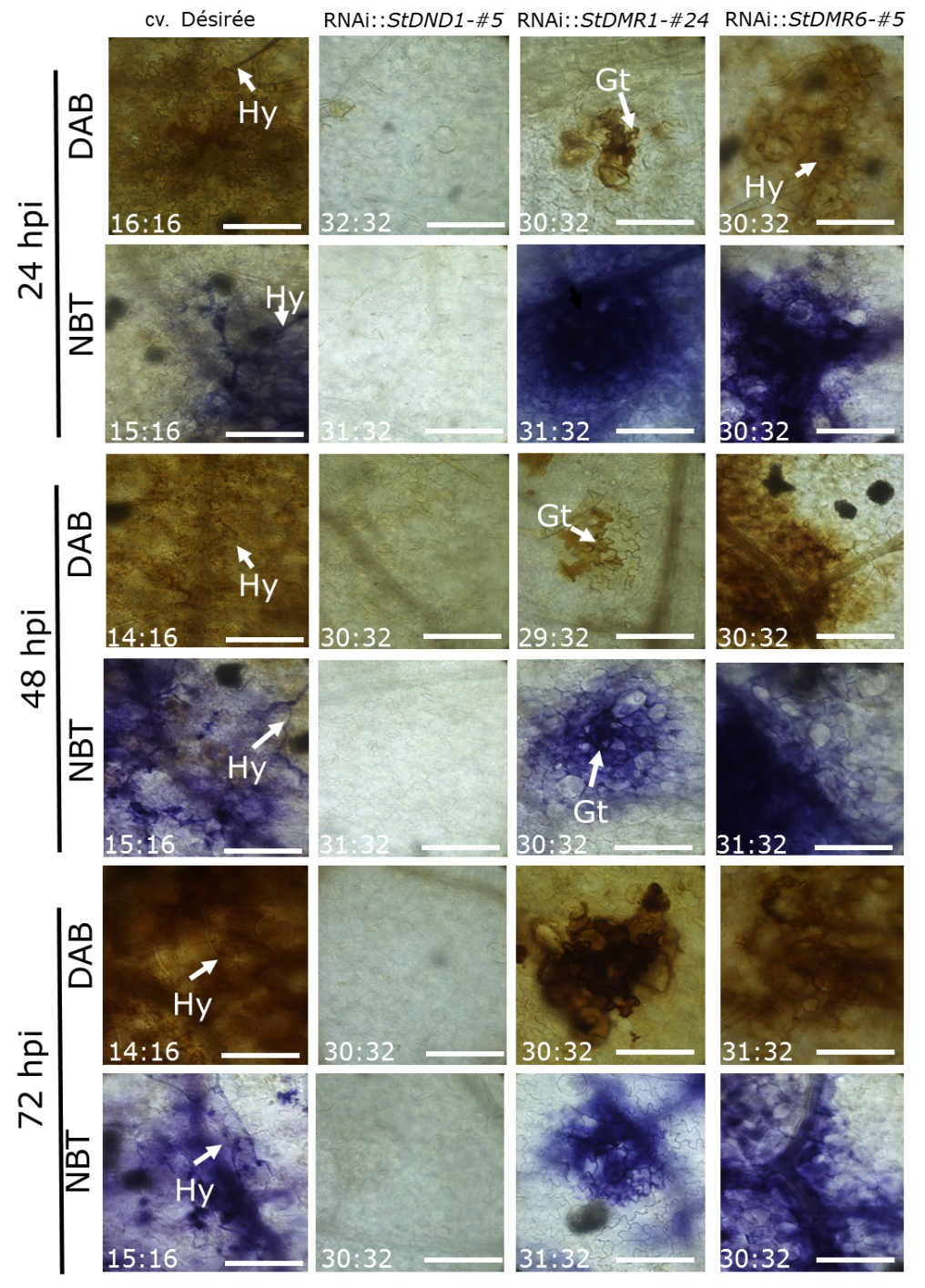
**

**Fig. S4.** **ROS accumulation on *S*-gene silenced potato plants.**

Microscopic images of potato leaves stained with DAB (H_2_O_2_) and NBT (O_2_^-^) at 24, 48 and 72 hpi with *P. infestans* EY6. Each image represents a single inoculation site. The numbers in each image represent the ratio of inoculated sites with similar responses and the total inoculated sites. Scale bars represent 100 μm. Germinated cyst (Gc), germ tube (Gt), hyphae (Hy).

**
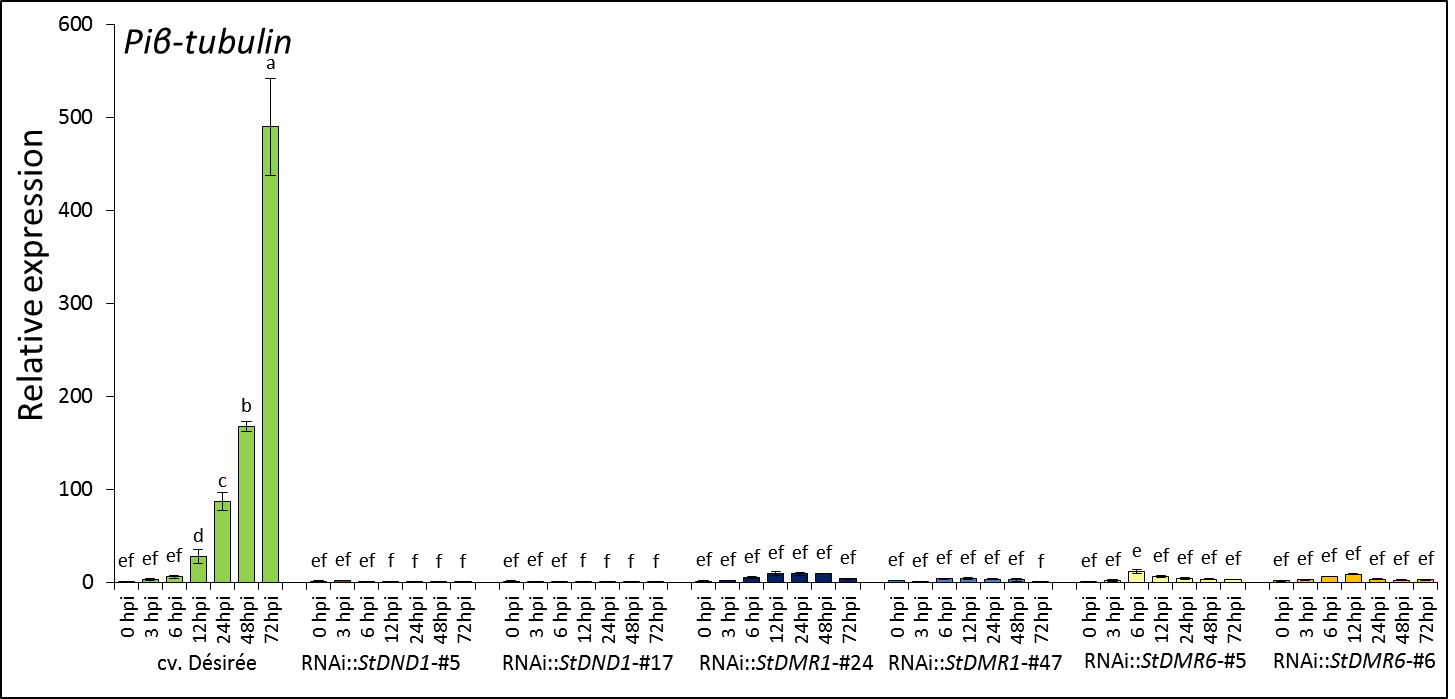
**

**Fig. S5. Relative expression of *Phytophthora infestans* nuclear gene *β-tubulin* in *S*-gene silenced potato lines upon infection (0-72 hours post inoculation (hpi)).** Transcript levels were normalized using the reference gene *EF1a* and expressed as genotype changes in the time-course experiment (0-72 hpi). Different letters indicate significant differences between time points according to Duncan’s multiple range test (P<0.05; n=3) performed in SPSS.

**
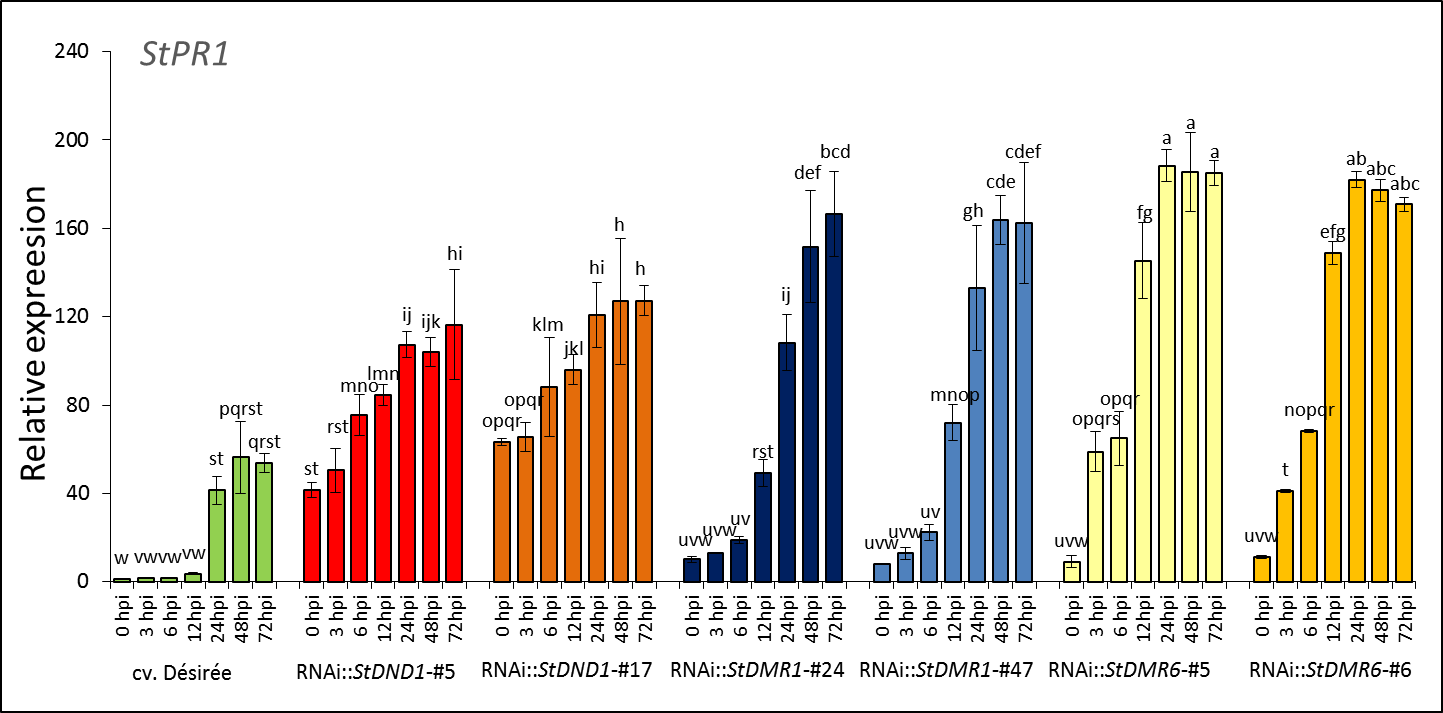
**

**
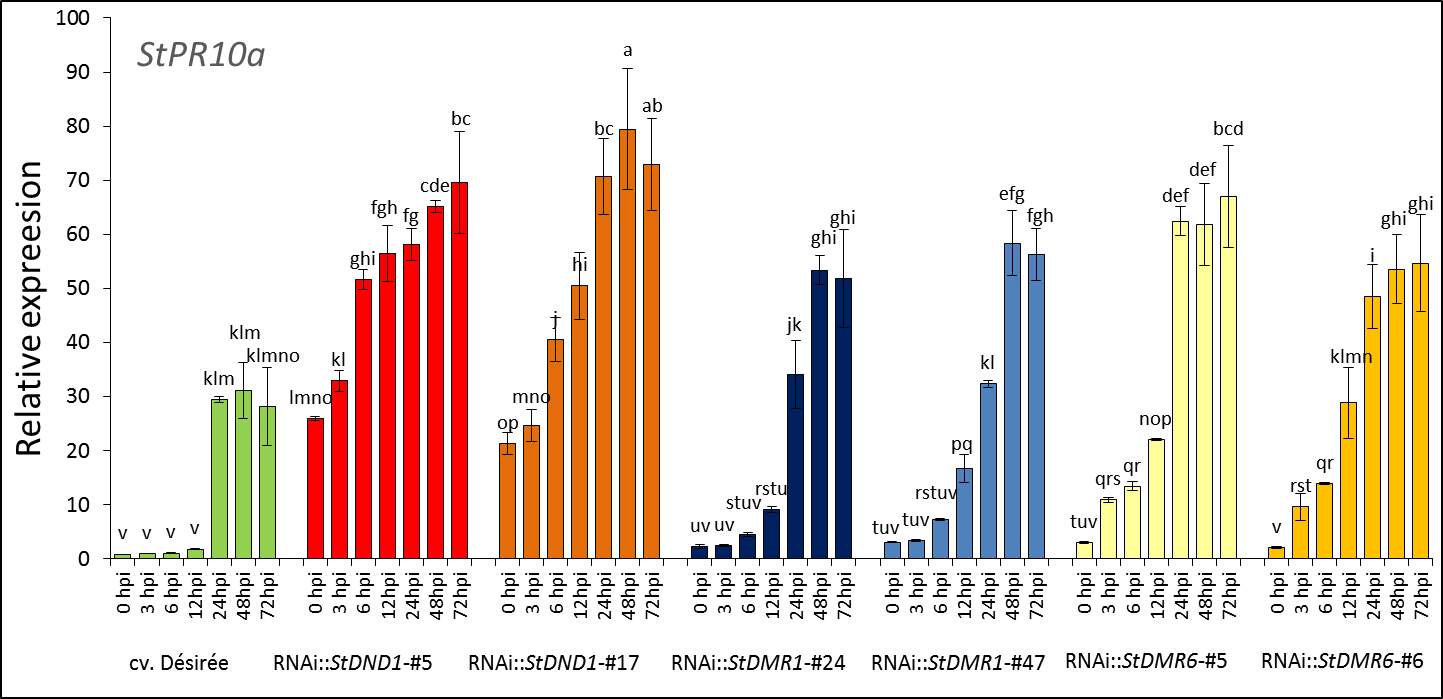
**

**
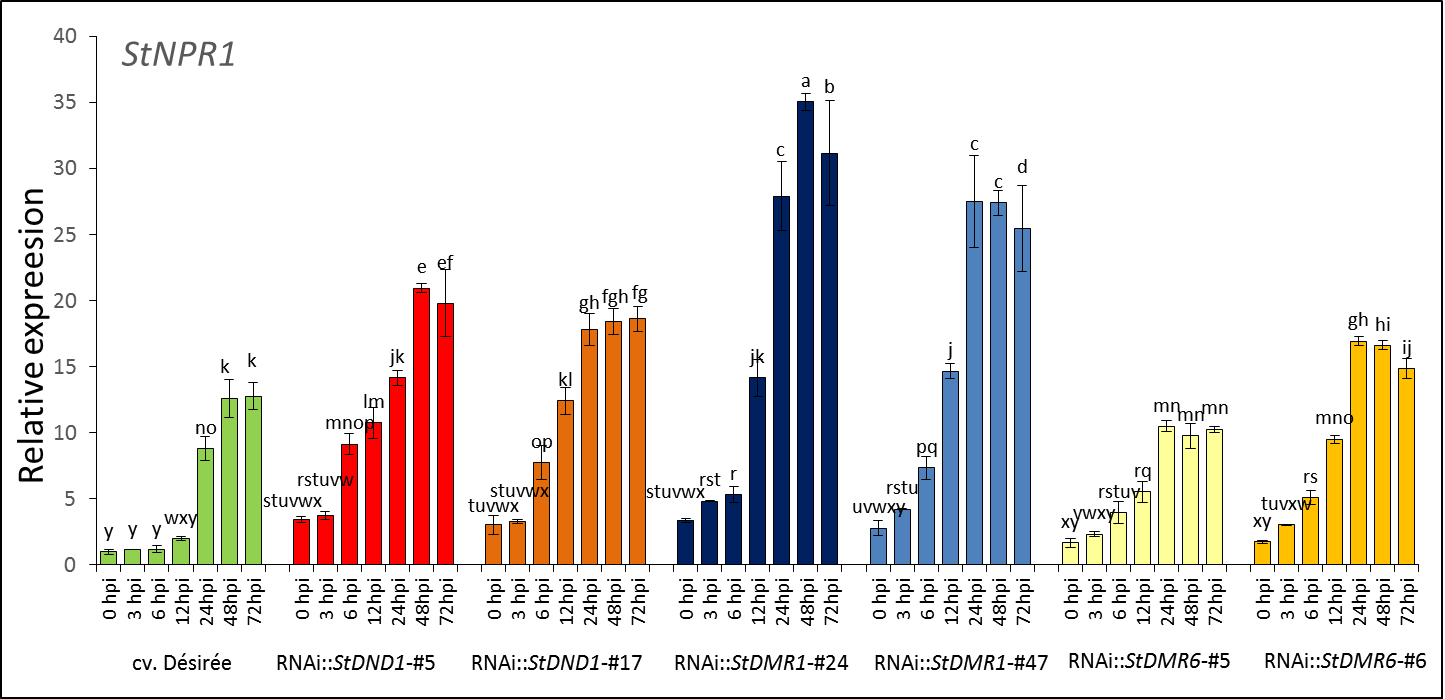
**

**
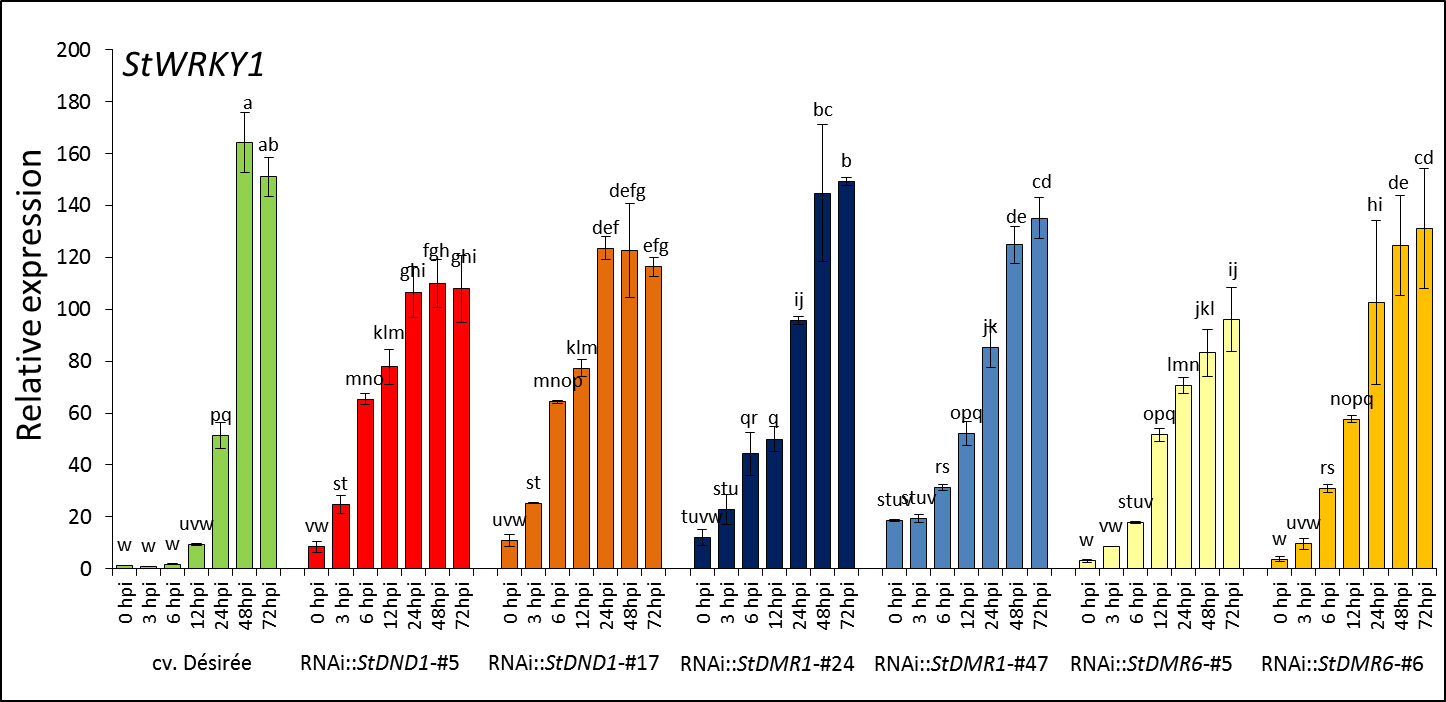
**

**
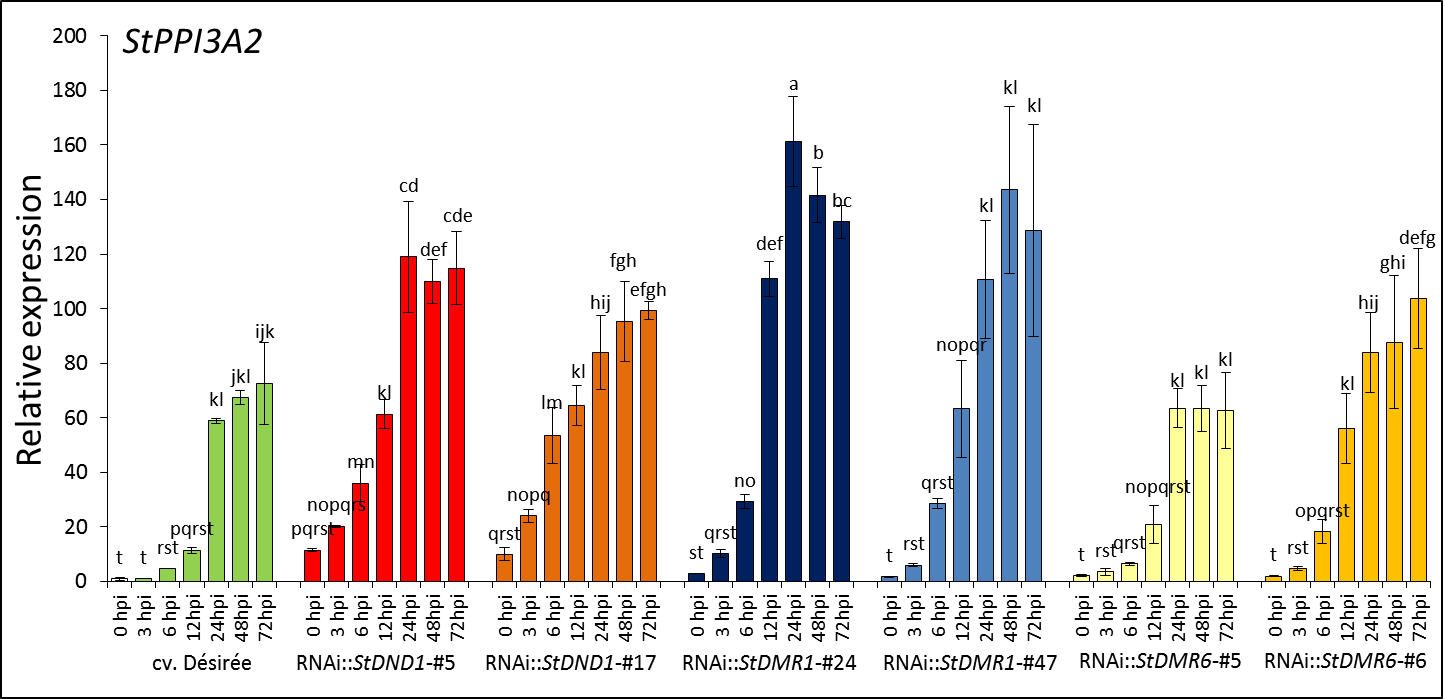
**

**Fig. S6. Relative expression of five salicylic acid pathway genes (*StPR1,* *StPR10a*, *StNPR1, StWRKY1* and *StPP13A2*) in *S*-gene silenced potato lines upon infection (0-72 hours post inoculation (hpi)).** Transcript levels were normalized using the reference gene *EF1a* and expressed as genotype changes in the time-course experiment (0-72 hpi). Different letters indicate significant differences between time points according to Duncan’s multiple range test (P<0.05; n=3) performed in SPSS.

**
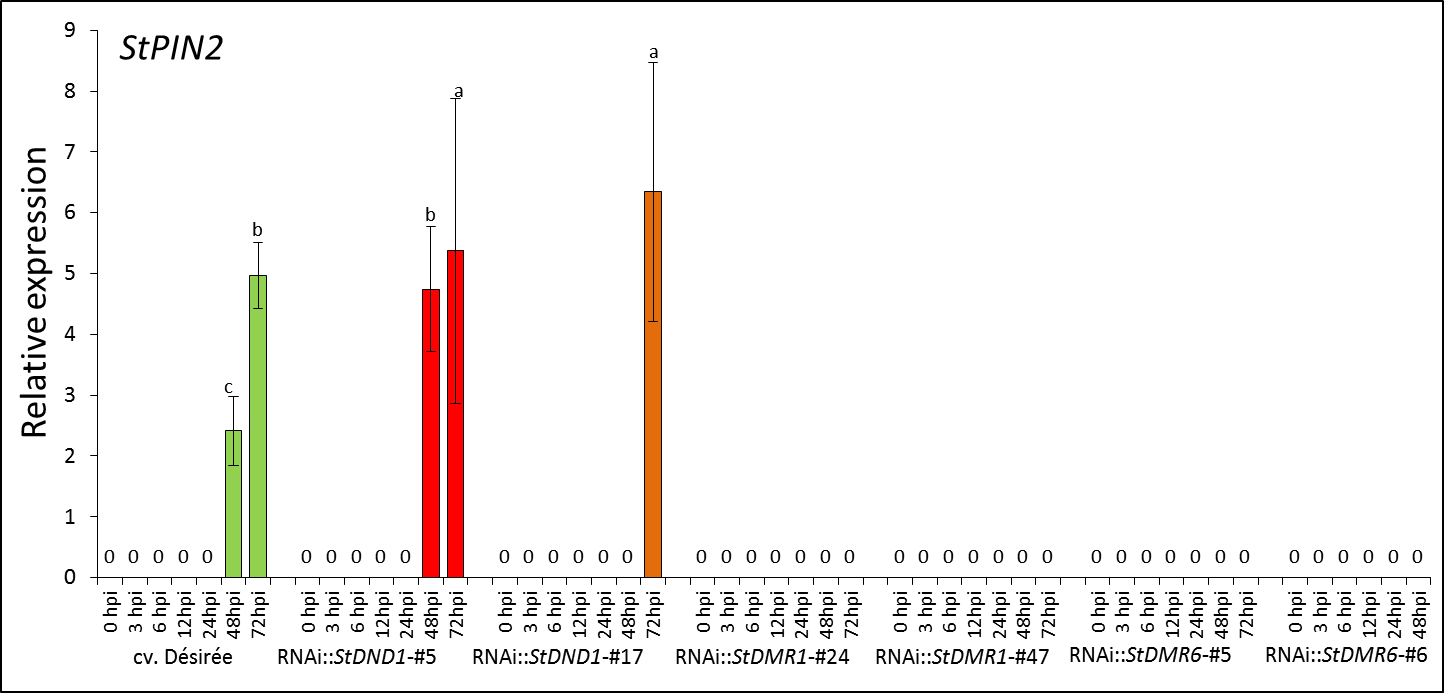
**

**Fig. S7. Relative expression of jasmonate pathway gene *StPIN2* in *S*-gene silenced potato lines upon infection (0-72 hours post inoculation (hpi)).** Transcript levels were normalized using the reference gene *EF1a* and expressed as genotype changes in the time-course experiment (0-72 hpi). Different letters indicate significant differences between time points according to Duncan’s multiple range test (P<0.05; n=3) performed in SPSS.

**
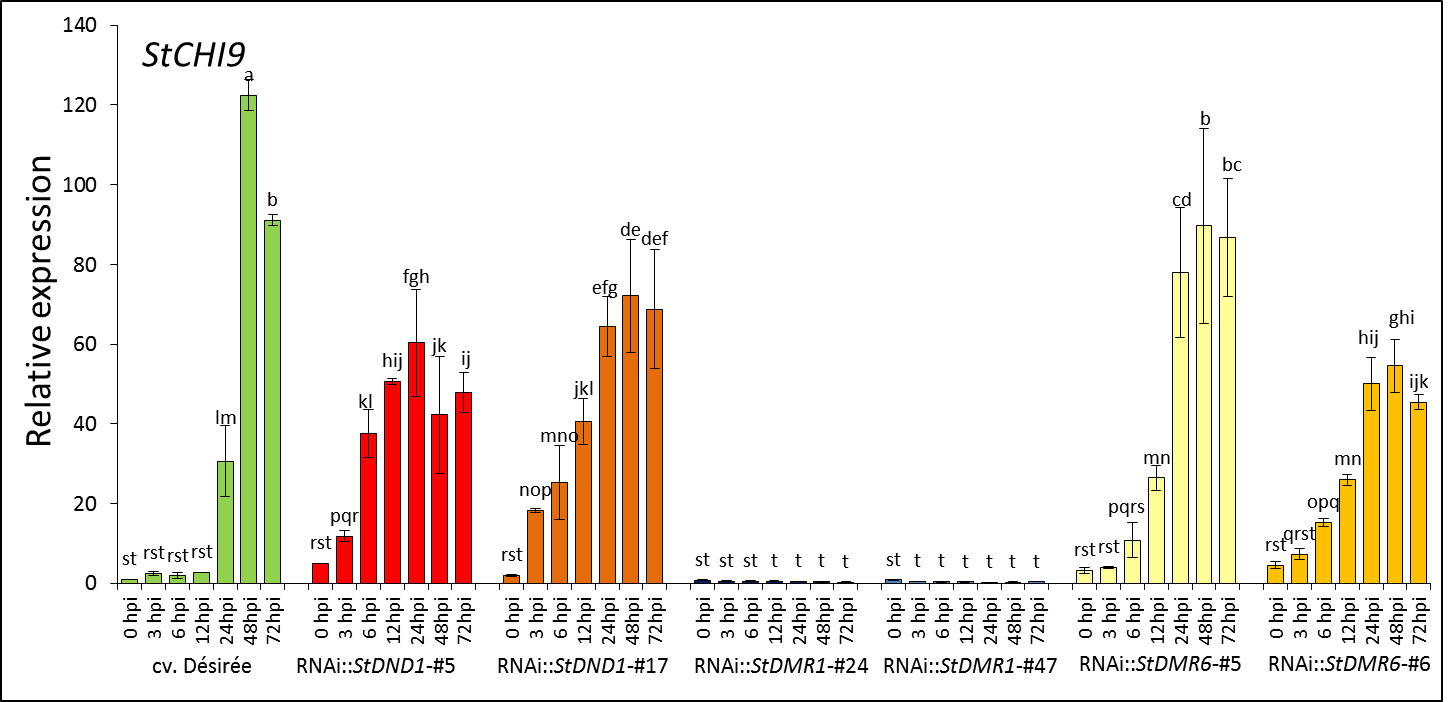
**

**Fig. S8. Relative expression of ethylene pathway gene *StCHI9* in *S*-gene silenced potato lines upon infection (0-72 hours post inoculation (hpi)).** Transcript levels were normalized using the reference gene *EF1a* and expressed as genotype changes in the time-course experiment (0-72 hpi). Different letters indicate significant differences between time points according to Duncan’s multiple range test (P<0.05; n=3) performed in SPSS.

**
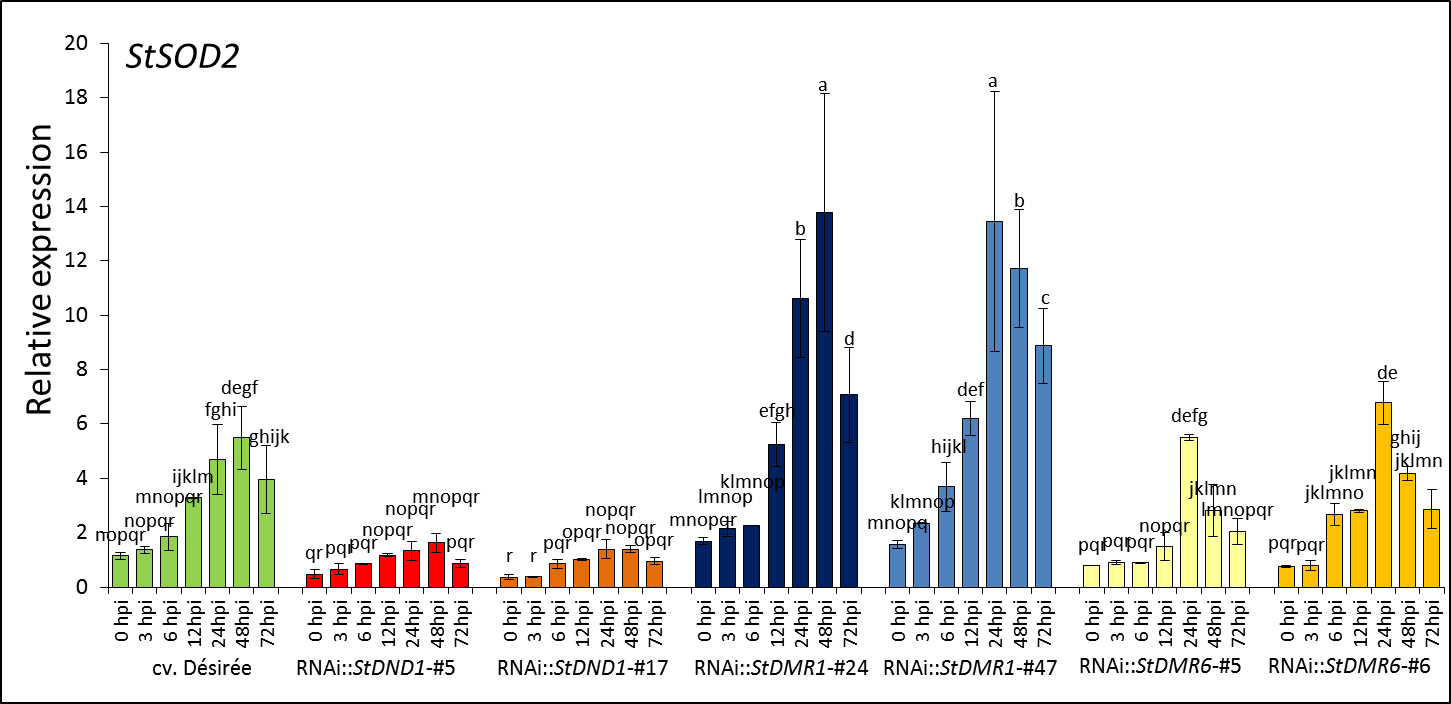
**

**
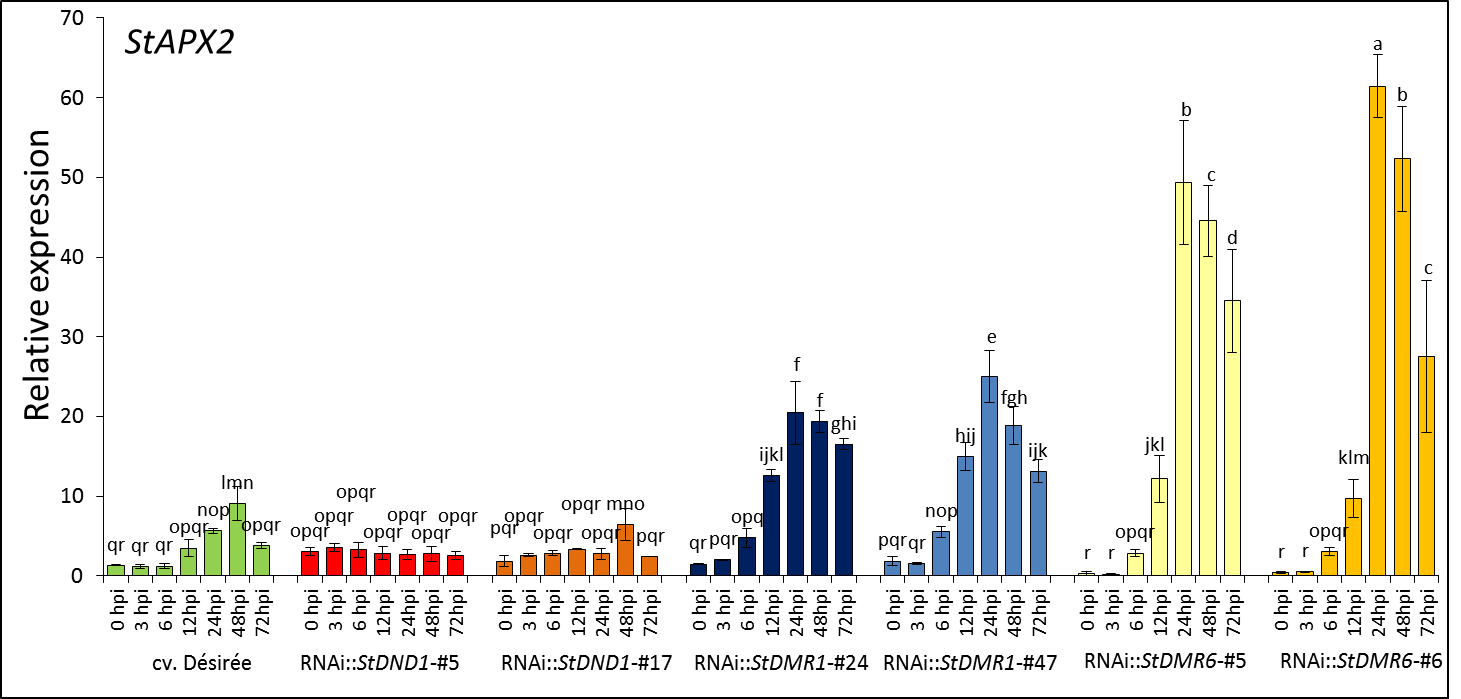
**

**
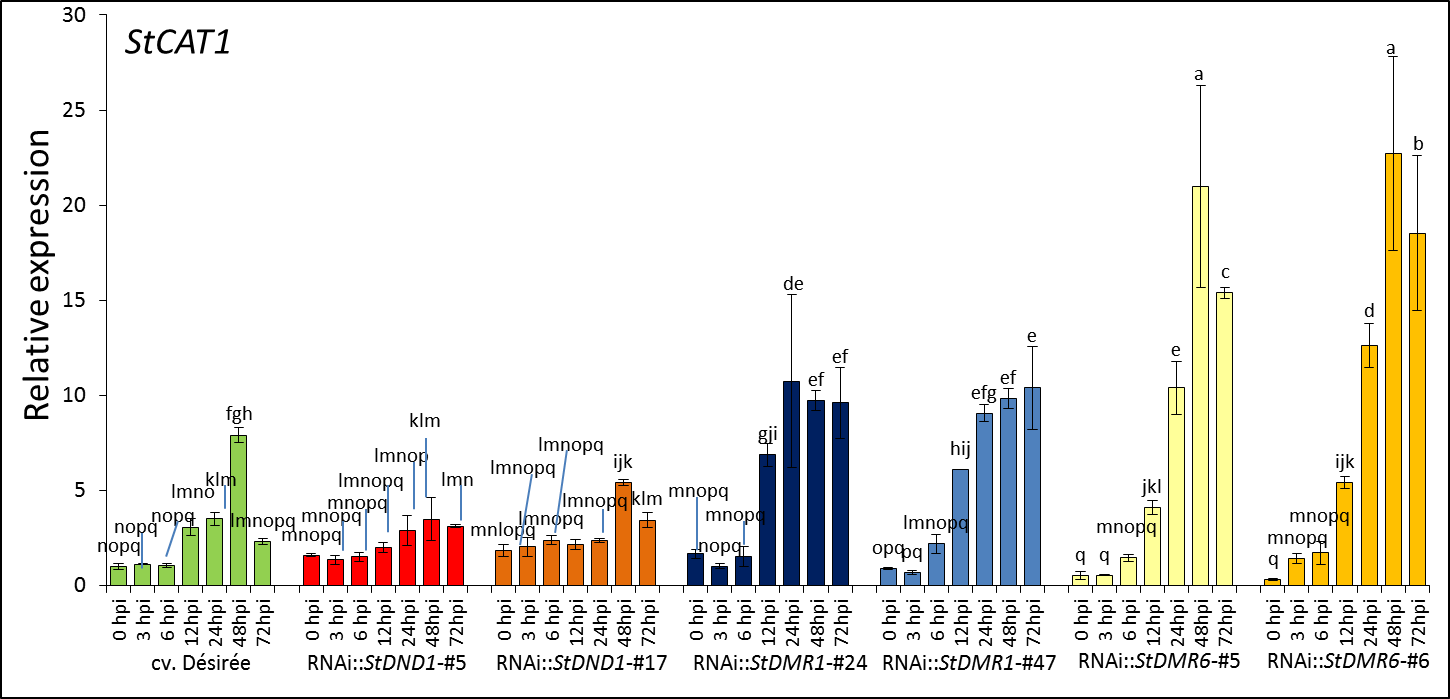
**

**Fig. S9. Relative expression of three oxidative burst-related genes (*StSOD2*, *StAPX2*, and *StCAT1*) in *S*-gene silenced potato lines upon infection (0-72 hours post inoculation (hpi)).** Transcript levels were normalized using the reference gene *EF1a* and expressed as genotype changes in the time-course experiment (0-72 hpi). Different letters indicate significant differences between time points according to Duncan’s multiple range test (P<0.05; n=3) performed in SPSS.

**
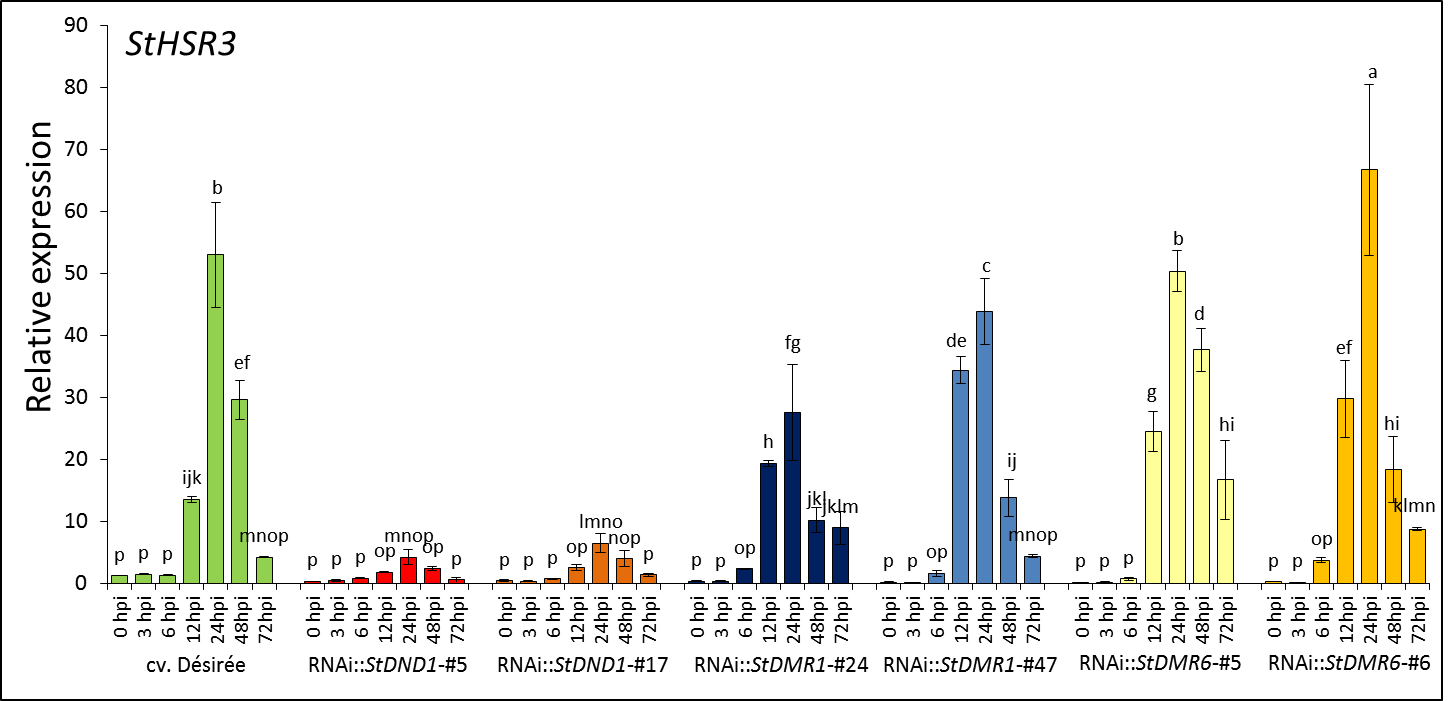
**

**
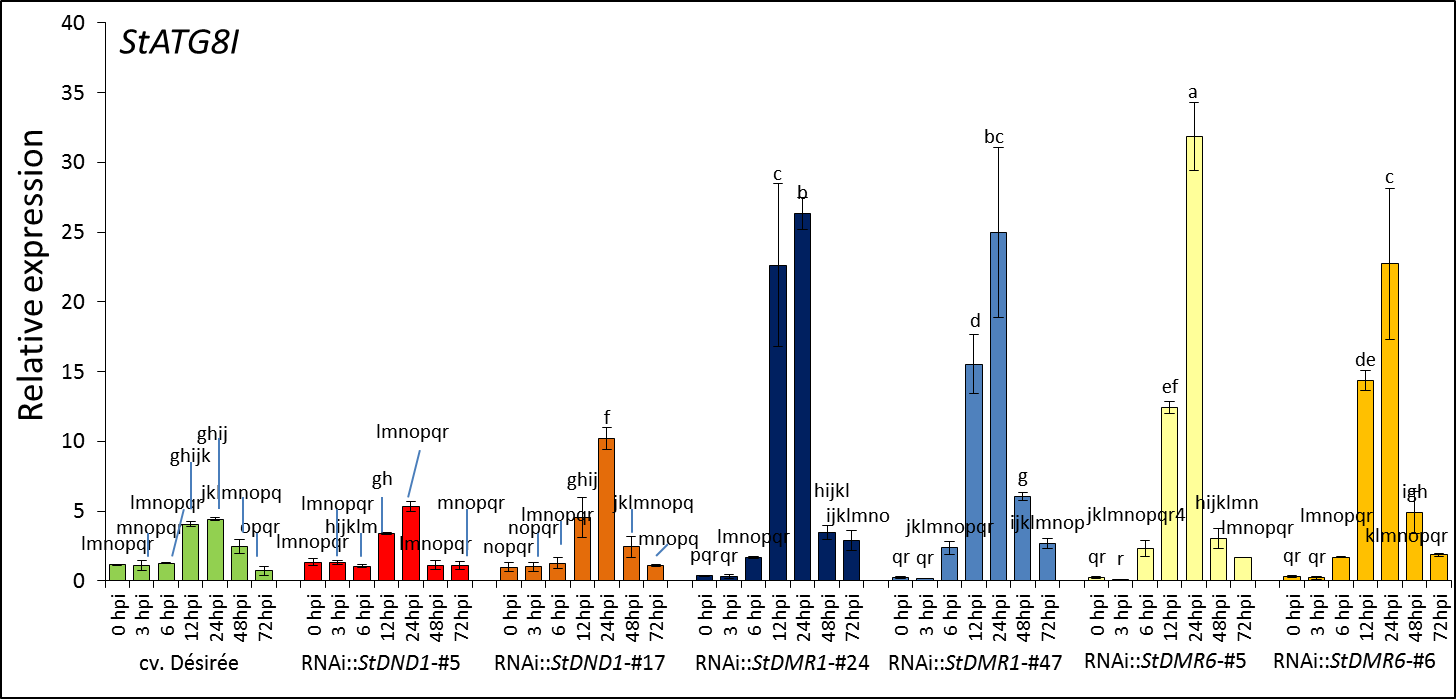
**

**Fig. S10. Relative expression of two cell death-related gene *StHSR3* and *StATG8I* in *S*-gene silenced potato lines upon infection (0-72 hours post inoculation (hpi)).** Transcript levels were normalized using the reference gene *EF1a* and expressed as genotype changes in the time-course experiment (0-72 hpi). Different letters indicate significant differences between time points according to Duncan’s multiple range test (P<0.05; n=3) performed in SPSS.
